# Improving the Accuracy of Bulk Fitness Assays by Correcting Barcode Processing Biases

**DOI:** 10.1093/molbev/msae152

**Published:** 2024-07-23

**Authors:** Ryan Seamus McGee, Grant Kinsler, Dmitri Petrov, Mikhail Tikhonov

**Affiliations:** Department of Physics, Washington University, St. Louis, MO, USA; Department of Bioengineering, University of Pennsylvania, Philadelphia, PA, USA; Department of Biology, Stanford University, Palo Alto, CA, USA; Department of Physics, Washington University, St. Louis, MO, USA

**Keywords:** fitness, sequence barcodes, high-throughput assays, systematic bias, inference

## Abstract

Measuring the fitnesses of genetic variants is a fundamental objective in evolutionary biology. A standard approach for measuring microbial fitnesses in bulk involves labeling a library of genetic variants with unique sequence barcodes, competing the labeled strains in batch culture, and using deep sequencing to track changes in the barcode abundances over time. However, idiosyncratic properties of barcodes can induce nonuniform amplification or uneven sequencing coverage that causes some barcodes to be over- or under-represented in samples. This systematic bias can result in erroneous read count trajectories and misestimates of fitness. Here, we develop a computational method, named REBAR (Removing the Effects of Bias through Analysis of Residuals), for inferring the effects of barcode processing bias by leveraging the structure of systematic deviations in the data. We illustrate this approach by applying it to two independent data sets, and demonstrate that this method estimates and corrects for bias more accurately than standard proxies, such as GC-based corrections. REBAR mitigates bias and improves fitness estimates in high-throughput assays without introducing additional complexity to the experimental protocols, with potential applications in a range of experimental evolution and mutation screening contexts.

## Introduction

**Fig. 1.**
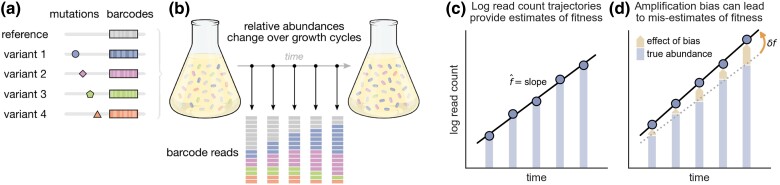
Genetic barcodes that are susceptible to systematic amplification and sequencing biases can cause misestimates of fitness. a) A standard approach for bulk fitness assays involves labeling a library of genetic variants with unique sequence barcodes. b) The variant library is pooled and grown in batch culture, often over multiple serial dilution growth cycles. Samples of the batch culture are taken at designated time points, and barcode sequences are extracted, amplified, and sequenced for each sample. The frequency of a barcode among all sequencing reads in a sample provides an estimate of the corresponding variant’s relative abundance in the batch culture at that time. c) The relative abundances of variants are expected to change exponentially over time according to their relative fitnesses. As such, the log read count of each variant’s barcode is expected to change linearly, where the slope of the best-fit line provides an estimate of the variant’s fitness $\bar{\;f}$ (i.e. exponential growth rate). d) However, bias-inducing factors in the amplification and sequencing process may cause barcode counts to be under- or over-represented relative to the true abundance of the corresponding variants in culture, which can lead to a misestimation of fitness, δf.

Standard assays for measuring microbial fitness involve tracking the abundances of variants over time in batch culture competitions and using these data to estimate relative growth rates (Wiser and Lenski 2015). High-throughput sequencing technology makes it possible to measure the fitnesses of many strains simultaneously using batch culture assays (Smith et al. 2009; Araya and Fowler 2011; Mehlhoff and Ostermeier 2020). In this approach, a collection of variants is labeled with unique sequence barcodes for identification (Fig. 1a). The pooled variant library is then competed against a reference strain (e.g. the ancestor) over multiple growth cycles (Fig. 1b). The batch culture is sampled at designated intervals, and barcode regions are extracted, amplified, and sequenced for each sample. The relative abundance of a variant at a given time can be determined from the fraction of the total sequencing reads that map to its barcode in the corresponding sample. In the simplest approach, the relative fitness of each variant can be estimated by fitting a linear model to its log-count trajectory (Fig. 1c).

This approach provides good estimates of fitness when barcode read counts are a reliable reflection of the relative abundances of variants in the culture. However, multiple factors can introduce variability in read counts. Some noise is expected due to stochasticity in growth cycles, perturbations in culture conditions (e.g. “batch effects” such as incubation temperature, nutrient concentrations, or inoculum densities), and bottlenecks associated with serial transfer and sampling. The uncertainty in fitness estimates attributable to random noise is, by definition, unavoidable, but can typically be quantified and mitigated through suitable control strategies.

Here we are concerned with sources of bias that cause barcode read counts to *systematically* deviate from the variants’ true culture abundances. While genetic barcodes are often assumed to be inert labels, it has been shown that their sequence properties can lead to nonuniform amplification and uneven coverage in next-generation sequencing pipelines (Smith et al. 2008; Aird et al. 2011; Thielecke et al. 2017). For example, the base composition of a barcode can modulate its sensitivity to small fluctuations in temperature ramps and enzyme activities during PCR amplification, which can cause some barcodes to be consistently over- or under-represented in samples (Benjamini and Speed 2012; Laursen et al. 2017; Johnson et al. 2023). This can introduce systematic biases that result in erroneous read counts and misestimates of fitness (Fig. 1d). Barcode representation bias is known to correlate with statistics such as GC ratio (Southern et al. 1999; Margulies et al. 2001; Dohm et al. 2008; Hillier et al. 2008; Benjamini and Speed 2012; Laursen et al. 2017), but the relationship between amplicon sequence and bias is more complex than GC content alone and has not been fully characterized (Kuo et al. 2002; Aird et al. 2011). Furthermore, amplification bias also depends on the idiosyncratic processing conditions for each sample, which makes it challenging to determine the extent to which counts and fitness estimates have been impacted by bias (Siddiqui et al. 2006).

One strategy to mitigate barcode-associated bias is to label each variant with multiple distinct barcodes such that their differing biases average out when taken as an ensemble (Ardell et al. 2023). However, the addition of redundant barcodes can introduce substantial complexity to library preparation, which limits the scalability of this approach. Beyond this, it is not straightforward to label variants with redundant barcodes in many experimental evolution contexts, such as in lineage tracking experiments where a clonal ancestral population is pre-labeled with random barcodes before de novo mutants are spontaneously generated (Levy et al. 2015; Venkataram et al. 2016).

Here we introduce a data-driven method, named REBAR (Removing the Effects of Bias through Analysis of Residuals), that infers and removes the effects of barcode processing bias by leveraging the structure of systematic error across an experimental data set. REBAR offers a procedure for improving high-throughput fitness estimates that does not require changes to protocols and can even be applied retroactively to existing data.

## A Data-Driven Approach

We aim to infer and correct systematic biases using the data that is typically collected in bulk fitness assays, namely barcode read counts for a library of variants measured across time series of competition culture samples. Often in such experiments, the same barcode-labeled library is assayed multiple times, either as replicates or to measure fitnesses in alternative environmental conditions. The involvement of the same barcodes in multiple assays enhances the accuracy of our bias inference, but our method can also be applied to single-assay data.

For narrative simplicity, we assume that sequencing depth (i.e. total read count) is the same for all samples, and we refer simply to “barcode counts” rather than “normalized counts” or “relative abundances” (supplementary Section S1.1, Supplementary Material online). Sequencing depth is never actually uniform, but correcting for varying sequencing depth is standard practice (in fact, our algorithm has a built-in capacity to do so; see supplementary Section S2.1b, Supplementary Material online). We further assume that the variants of interest make up a small fraction of the batch culture relative to the reference strain, such that their change in abundance during an assay is well-modeled by an exponential. Making this assumption and accounting for deviations from it are also standard practice. Under these assumptions, the log-counts of each barcode are expected to follow linear trajectories, which simplifies the presentation of our approach.

### Model of Underlying Bias

In practice, observed counts reflect not only the relative abundances of variants but also the effects of noise and bias that arise in the barcode amplification and sequencing process. Here we are concerned with the contributions of barcode processing bias in particular. This bias differs from random noise in that its effects on observed counts will tend to impact a given barcode in a systematic way across samples. That is, a variant whose barcode is highly susceptible to bias will tend to have its counts affected more strongly across all samples, compared to other variants. Similarly, samples with procedural conditions that induce substantial bias will exhibit greater impacts on variants’ counts across the board when compared to other samples.

We model the effect of bias $b_{i,t}^{\alpha }$ on the observed count of variant *i* at time *t* in assay *α* as the product of the variant’s characteristic susceptibility to bias $u_{i}$ and the prevalence of bias in that sample $v_{t}^{\alpha }$:


(1) $$b_{i,t}^{\alpha }=u_{i}v_{t}^{\alpha }.$$

**Fig. 2.**
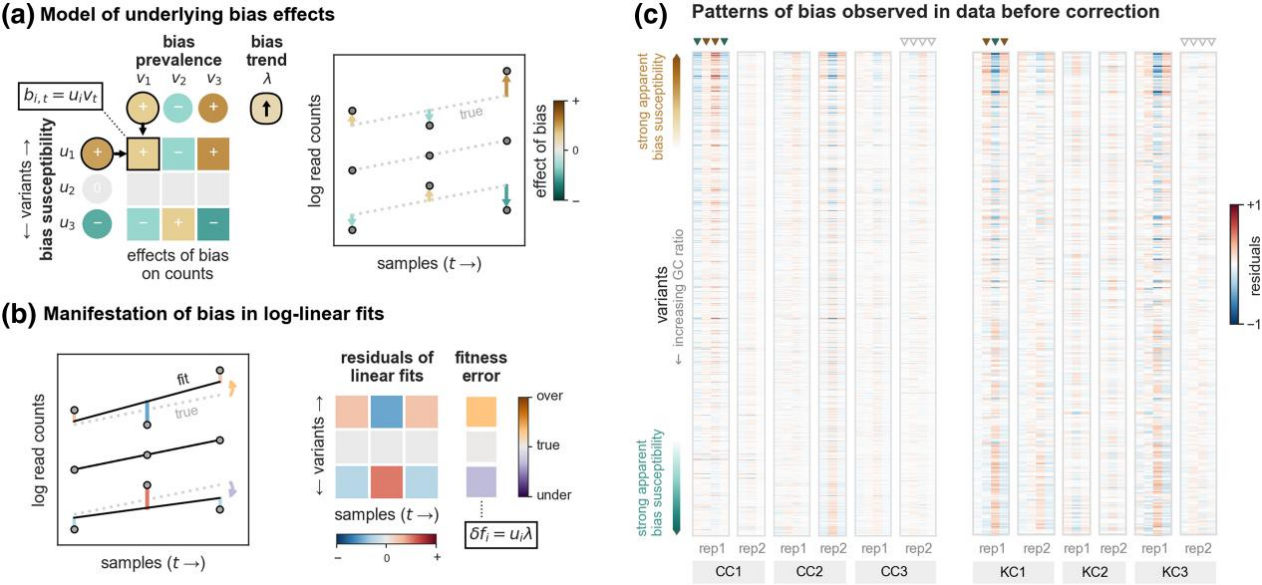
Barcode processing bias impacts the structure of residuals and the accuracy of fitness estimates derived from linear fits of log-count trajectories. a) Log-count trajectories are shown for a hypothetical fitness assay with three variants (right). The contributions of barcode processing bias to the observed counts are shown in the table (left). We model the effect of bias on the count for variant *i* in sample *t* as the product of the prevalence of bias in the sample ($v_{t}$ values, one per sample) and the variant’s susceptibility to bias ($u_{i}$ values, one per variant). These bias effects cause observed counts (points, right) to deviate (arrows, right) from the log-linear trajectories that correspond to each variant’s true change in abundance over time (dotted lines, right). b) A line is fit to each variant’s observed log-count trajectory (black lines, left). The residuals of each fit are depicted as bars (left) and in the table (right). The effects of underlying bias are reflected in the structure of the magnitudes and signs of residuals (refer to the color scale below the table). Note the similarities between the bias effect table in a) and the residuals table in (b). In this example, bias prevalence has a slight positive trend across time points (see $v_{t}$ and *λ* values in (a)), which causes the slope of each variant’s log-linear fit to deviate from that of its true trajectory as modulated by its bias susceptibility (arrows, left), which results in misestimates of fitness (column, far right). c) Heatmaps depict the residuals of linear fits to log-count trajectories from two published bulk fitness assay data sets: Chen et al. (2023) (left) and Kinsler et al. (2020) (right). For each data set, we show residuals for a library of variants (rows) across six fitness assays conducted in three different environmental conditions (“Chen conditions” CC1, CC2, and CC3; and “Kinsler conditions” KC1, KC2, and KC3; see supplementary Section S3, Supplementary Material online, for more information). Variants (rows) are ordered by the GC ratio of their barcodes, increasing from top to bottom. Barcodes with the highest and lowest GC ratios tend to have large residuals across multiple samples, which is consistent with these variants being more susceptible to bias. Samples (individual columns) with relatively large residuals and a strong correlation between residuals and GC ratios are consistent with high-bias prevalence (example samples marked by solid arrows above table). By contrast, in other samples (such as those marked by open arrows above table), residuals are relatively uncorrelated with GC ratio, which indicates that bias prevalence is likely weak.

The bias prevalence component gives the overall strength and direction of bias in a sample, which reflects the tendency of the procedural conditions in that sample to influence barcode counts. In this model, the susceptibility of a variant’s barcode modulates how much that variant’s counts are affected by processing bias. Prevalent bias translates to relatively large shifts in counts for highly susceptible variants (Fig. 2a, top and bottom variants), while variants with negligible susceptibility are weakly affected by bias-inducing factors, even when they are highly prevalent (Fig. 2a, middle variant). The counts of “positively susceptible” barcodes are influenced in the opposite direction from “negatively susceptible” barcodes with respect to over- versus under-representation (Fig. 2a, compare top and bottom variants).

### Manifestation of Bias in Residuals and Misestimates of Fitness

The log-count of each variant’s barcode is expected to change linearly with a slope that corresponds to its growth rate. Fitting a line to the log-count trajectory of each barcode provides an estimate of the relative fitness ${\bar{\;f}}_{i}$ for each variant (Fig. 1c). However, barcode processing biases cause read counts to deviate from the “true” trajectories. Under our model of bias, we expect these effects to leave two notable signatures in the data.

The first effect is to perturb observed counts away from tightly log-linear trajectories. As a result, the residuals of a variant’s log-linear fit can reflect the magnitudes and directions of bias effects in the respective samples (note the correspondence between the underlying bias effects in Fig. 2a with and the observed residuals in Fig. 2b). Barcodes that are highly susceptible to bias often have large residuals in samples where bias is prevalent (Fig. 2b, top and bottom variants), whereas barcodes that are not susceptible to bias will be immune from these deviations (Fig. 2b, middle variant).

We checked for these patterns of bias-driven residuals in two published bulk fitness assay data sets, namely Chen et al. (2023) and Kinsler et al. (2020) (Fig. 2c). Both studies collected barcode read count time series for hundreds of yeast variants across a number of assay conditions (a subset of which are shown in Fig. 2c; see supplementary Section S3, Supplementary Material online, for more information). We see that both data sets contain samples that exhibit strong residuals where the sign and magnitude of residuals also appear to be correlated with the GC ratio (i.e. row ordering) of the respective barcodes (e.g. columns marked by solid arrows in Fig. 2c). This structure is consistent with systematic barcode processing bias being prevalent in those samples, in contrast to other samples where residuals are more random across variants (e.g. columns marked by open arrows in Fig. 2c). Variants that have large residuals in one sample tend to have relatively large residuals in other samples as well, suggesting that these variants are more susceptible to bias-inducing effects. Those variants that appear to be most susceptible to bias (i.e. those that have large residuals across samples) tend to have among the highest or lowest GC ratios in their respective library (i.e. are near the top or bottom of the heatmaps in Fig. 2c), but the correlation between apparent bias susceptibility and GC ratio is imperfect. This is expected because barcode processing bias is related to a number of sequence properties, with GC content being just one factor.

The second major effect of bias is to cause the best-fit slope of a variant’s log-count trajectory to deviate from its actual rate of change in abundance (Fig. 2b). This occurs when the effects of bias vary from one sample to the next with a non-zero trend over time (such as in Fig. 1d). Such correlation of bias prevalence with time can arise by chance and is more likely to occur when the number of sample time points per assay is small, as is often the case. A temporal trend in bias will confound the signal from a variant’s change in abundance and result in misestimates of fitness (note that bias with a constant effect on all counts over time shifts the log-linear fit vertically but does not change its slope). The degree to which a variant’s fitness estimate is impacted by a trend in bias prevalence is modulated by its bias susceptibility (Fig. 2b).

We can formalize the temporal trend of bias prevalence in an assay with a linear model


(2) $$v_{t}^{\alpha }=\lambda^{\alpha }t+\gamma_{t}^{\alpha },$$

where $\lambda^{\alpha }$ gives the change in prevalence over time and $\gamma_{t}^{\alpha }$ is the incidental deviation from this linear trend for each individual sample. Then, the error in a variant’s fitness estimate relative to its true fitness (i.e. $\delta {\;f}_{i}^{\alpha }={\bar{\;f}}_{i}^{\alpha }-{\;f}_{i,\mathrm{true}}^{\alpha }$) is given by the product of the variant’s bias susceptibility and the trend in bias prevalence in that assay (supplementary Section S1.3, Supplementary Material online):


(3) $$\delta f_{i}^{\alpha }=u_{i}\lambda^{\alpha }.$$

It is impossible to disambiguate bias-driven trends in counts from fitness-driven ones using counts or residuals alone. Nevertheless, as we explain in the next section, having just *one* control group of variants with equal true fitnesses is sufficient to fully resolve this problem and infer the effects of bias for an entire library.

### Disentangling Bias Trends from Fitness Estimates

**Fig. 3.**
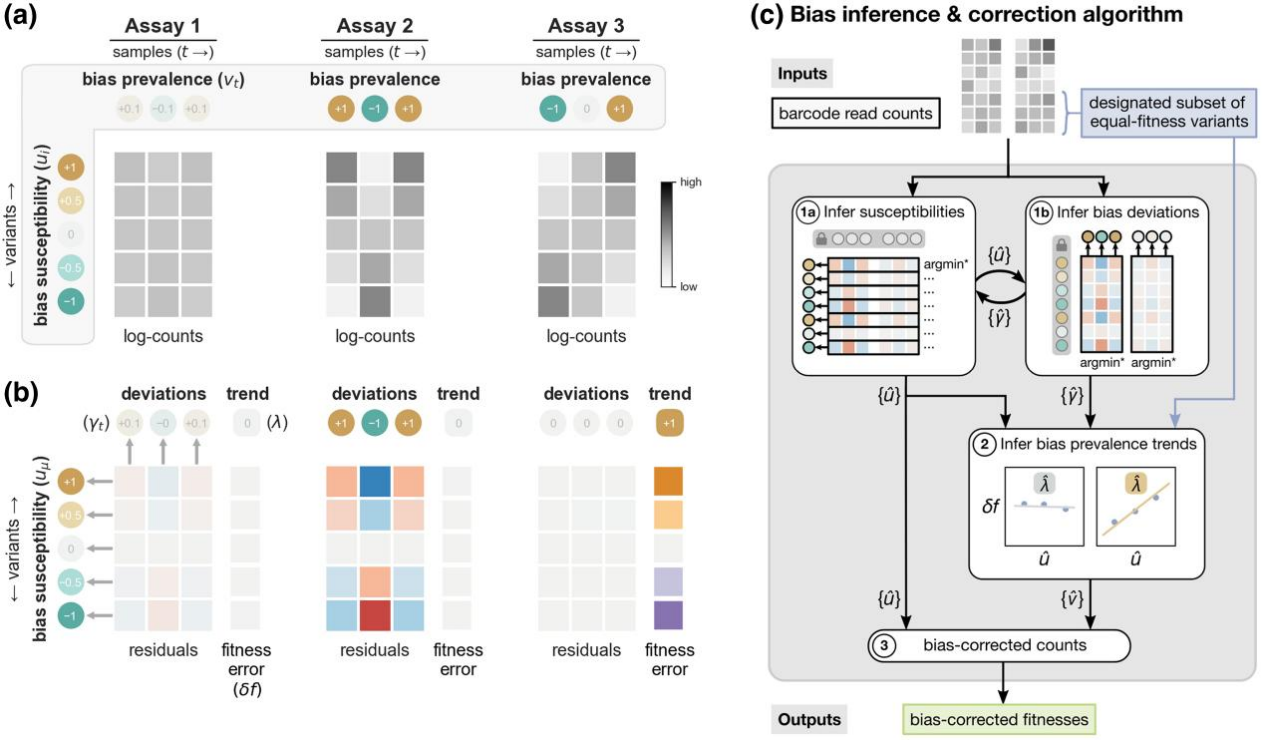
Decomposing bias into trend and deviation components enables their inference. a) Log-counts from three hypothetical assays involving a set of five variants are shown (gray-scale tables). For illustrative purposes, these five variants are assumed to all have the same fitness as the reference strain. The true abundances of these variants remain constant in culture, but observed counts deviate from constant trajectories due to the effects of barcode processing bias. The “ground truth” bias susceptibility and bias prevalence components that give rise to the observed counts are shown in the gray outlined region. b) The residuals of linear fits to the trajectories in (a) are depicted in the lower tables for each assay. The errors in fitness estimates due to bias-induced shifts in log-count trajectories are given in the columns to the right of each residuals table. These linear fits effectively decompose counts data into trends (fitnesses) and deviations (residuals). Decomposing bias prevalence into trend (*λ*) and deviation ($\gamma_{t}$) components as well enables us to infer these components using the analogous terms derived from the counts data (see the text for more information). c) A graphical schematic of the two-stage bias inference algorithm is shown (see the text and supplementary Section S2, Supplementary Material online, for more information).

Consider the thought experiment presented in Fig. 3, where the ground truth fitnesses, bias susceptibilities, and sample bias prevalences underlying a three-assay data set are known. Here, we consider a set of neutral variants that all have the same fitness as the reference strain (e.g. the ancestor). For such a set, the true relative abundances remain constant throughout each assay, but the barcode read counts (shown as gray-scale tables in Fig. 3a) exhibit fluctuations due to the effects of bias in each sample.

Overall, each variant’s pattern of residuals across all assays (i.e. the signs and magnitudes in each row of residuals) tends to correspond to the strength and direction of that variant’s underlying susceptibility. Similarly, the underlying bias prevalence values are often associated with a matching pattern of residuals across variants in the respective columns.

However, the relationship between bias prevalence and residuals is more nuanced, as is illustrated when comparing the three assays in this example. In Assay 1, bias prevalence is low in all samples, all residuals are correspondingly small, and fitnesses are accurately estimated. In Assay 2, bias prevalence is greater, and susceptible variants are more impacted as seen in larger residuals for those variants. However, fitness estimates remain accurate because the bias does not have a temporal trend that confounds variant growth over time (i.e. $\lambda^{\left(1\right)}=0$). Compare these outcomes with those of Assay 3, where bias prevalence increases linearly over time ($\lambda^{\left(3\right)}=1$). This results in log-linear count trajectories that are perfectly fit by lines with zero residuals (in the absence of other noise), but the apparent slopes are entirely due to the trend in bias and give erroneous fitness estimates. This illustrates why residuals alone are not sufficient to infer bias prevalence without additional information about the bias trend for each assay.

Notice that we have decomposed both log-counts and bias prevalence values into trend (slope) and deviation (residual) terms (Fig. 3b). Sets of residuals across variants in a sample are informative about the respective *deviation* of that sample’s bias prevalence from the overall trend in prevalence for the assay (note the correspondence between columns of residuals and bias prevalence deviation values, $\gamma_{t}^{\alpha }$, for all assays in Fig. 3). It follows that the observed trajectory slopes provide information about *trends* in bias prevalence.

Equation (3) tells us that we can quantify the trend in bias prevalence $\lambda^{\alpha }$ by regressing fitness errors $\delta f_{i}^{\alpha }$ against bias susceptibilities $u_{i}$. In general, it is unrealistic to assume that the fitness errors $\delta f_{i}^{\alpha }$ could be known, since the purpose of the assay is to measure fitnesses in the first place. However, if a subset of variants is known beforehand to have the same fitness (e.g. a set of genetically identical strains labeled with different barcodes), then, for that subset, fitness errors can be approximated by comparing the estimated fitness of each variant to the mean estimate of the group. Therefore, an assay’s trend in bias prevalence can be estimated using the fitness errors and bias susceptibilities obtained for this special subset. In this way, a *single subset* of equal-fitness variants provides the information necessary to infer the trends in bias that are experienced by *all* variants in the library.

Altogether, the logic of the REBAR method for inferring bias is as follows: (i) the magnitudes and signs of a variant’s collection of residuals across all available samples inform the magnitude and sign of that variant’s bias susceptibility; (ii) the magnitudes and signs of a sample’s collection of residuals over all variants inform the magnitude and sign of that sample’s deviation in bias prevalence from a general trend in prevalence over the respective assay; and (iii) the correlation of fitness misestimation with bias susceptibility among a control subset of equal-fitness variants informs the trend in bias prevalence itself. The inferred bias components are used to compute bias-corrected counts that no longer include spurious trends that confound estimates of fitness.

### The Bias Inference and Correction Algorithm

REBAR infers underlying bias components from barcode read count time series data in two stages.

First, we infer bias susceptibility values and bias prevalence deviation values using an iterative optimization process. Let $\log\;C_{i,t}^{\alpha }$ denote the observed log-count of variant *i* in sample *t* of assay *α* (supplementary Section S1.1, Supplementary Material online). Then, a particular set of bias susceptibility and bias prevalence estimates (${\hat{u}}_{i}$ and ${\hat{v}}_{t}^{\alpha }$, respectively) yields a corresponding set of bias-adjusted log-counts $\log\;A_{i,t}^{\alpha }$ for each variant-sample (supplementary Section S1.4, Supplementary Material online):


(4) $$\log\;A_{i,t}^{\alpha }=\log\;C_{i,t}^{\alpha }-{\hat{u}}_{i}{\hat{v}}_{t}^{\alpha }.$$

Each adjusted count time series can be fit by a log-linear model


(5) $$\log\;A_{i,t}^{\alpha }={\tilde{\;f}}_{i}^{\alpha }t+{\tilde{c}}_{i}^{\;\alpha }+{\tilde{r}}_{i,t}^{\alpha },$$

where ${\tilde{\;f}}_{i}^{\alpha }$ gives the adjusted fitness estimate for variant *i* in assay *α*, ${\tilde{c}}_{i}^{\;\alpha }$ gives the *y*-intercept, and ${\tilde{r}}_{i,t}^{\alpha }$ denotes the residual of each sample *t* from the log-linear fit (supplementary Section S1.4, Supplementary Material online). We employ the heuristic that accurate estimates of bias susceptibility and prevalence deviations will yield bias-corrected counts that minimize residuals across the data.

We begin by initializing bias susceptibility and prevalence terms to random values (supplementary Section S2.0, Supplementary Material online). To infer the bias susceptibility of variant *i*, we fix the set of bias prevalences at their current values and solve for the susceptibility value ${\hat{u}}_{i}$ that minimizes the set of residuals associated with that variant across all assays and samples in the data set (Fig. 3c, box 1a). Evaluating this optimization for every variant in turn produces an updated set of bias susceptibility values for the library. Next, we fix the set of variant susceptibilities at their current values and infer the bias prevalence deviation terms ${\hat{\gamma }}_{t}^{\alpha }$ that minimize the set of residuals in each assay *α* across all variants (optimization of bias prevalence deviation values is done on a per-assay basis; Fig. 3c, box 1b). We alternate between optimizing bias susceptibility values (given the current prevalence deviation values) and optimizing bias prevalence deviation values (given the current susceptibility values) until suitable convergence is reached (regularized objective functions are used to resolve symmetries and ensure convergence; see supplementary Section S2.1, Supplementary Material online).

In the second stage of our process, we infer the trend in bias prevalence for each assay in order to determine absolute estimates of bias prevalence. Following the logic derived from Equation (3), we estimate the trend in bias prevalence in an assay *α* by regressing fitness misestimates $\delta f_{i\in G}^{\alpha }$ against inferred bias susceptibilities ${\hat{u}}_{i\in G}$ for a group of variants *G* where these values are known (Fig. 3c, box 2). In particular, we perform this step using a designated control subset of variants that are known beforehand to have the same fitness, which allows us to estimate their fitness errors relative to the group mean. The slope of this regression provides an estimate of the trend in bias prevalence for the respective assay (see supplementary Figs. S7 and S11, Supplementary Material online, for examples). With inferred values for both the trends and deviations in bias prevalence in hand, we then compute absolute bias prevalence estimates ${\hat{v}}_{t}^{\alpha }$ for each sample (supplementary Section S2.2, Supplementary Material online).

The inferred bias susceptibility and bias prevalence values we obtain from this procedure allow us to estimate and correct the effect of bias for each individual count in the data set (Equation (4)). More information about the implementation of this algorithm is provided in supplementary Section S2, Supplementary Material online.

## Results

**Fig. 4.**
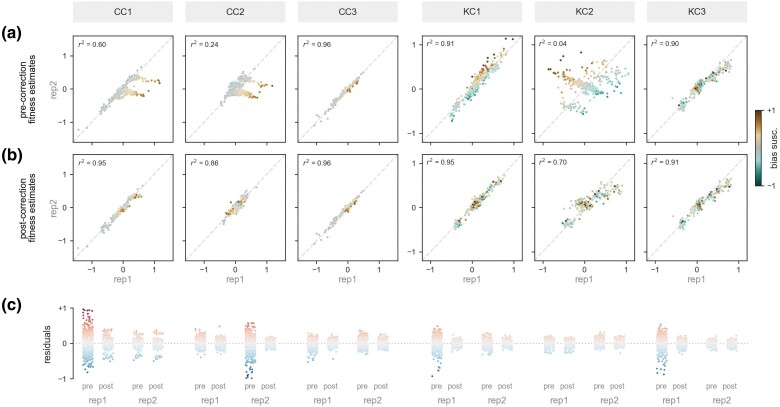
REBAR infers bias corrections that improve the accuracy of fitness estimates library-wide. a) Replicate fitness estimates are plotted for all variants (points) before bias correction for three-assay conditions each from Chen et al. (2023) and from Kinsler et al. (2020) (column headers: “Chen Conditions” CC1, CC2, and CC3; and “Kinsler Conditions” KC1, KC2, and KC3). Large discrepancies in fitness estimates between replicates suggest that fitness may be misestimated due to bias in one or both assays. Indeed, the magnitude of the replicate–replicate discrepancy (distance from 45-degree line) for a given variant is correlated with its bias susceptibility as inferred by REBAR (color scale). b) REBAR improves the accuracy of fitness estimates as seen in the tight correspondence of fitness estimates across replicates in all conditions after correction. c) REBAR’s corrections improve the log-linearity of count trajectories as seen in tight distributions of residuals (each point represents a variant-sample) for all assays after correction.

We applied REBAR to the bulk fitness assay data sets from Chen et al. (2023) and Kinsler et al. (2020) that were introduced above (Fig. 2c). Both data sets include barcode read count time series for a large library of yeast variants (2,586 and 548 variants, respectively) across multiple assay conditions (9 and 15 conditions, respectively; see supplementary Section S3, Supplementary Material online, for more information), and both libraries include a subset of near-equal-fitness variants suitable for use as the control set in REBAR (288 and 159 control variants, respectively; see supplementary Section S3, Supplementary Material online, for more information). Figure 4 summarizes the results of REBAR for replicate assays performed in three conditions from each study (“Chen conditions” CC1, CC2, and CC3; and “Kinsler conditions” KC1, KC2, and KC3), each of which highlights different features of REBAR (complete results are presented in supplementary Section S3, Supplementary Material online).

The objective of REBAR is to improve the accuracy of fitness estimates by removing the confounding effects of barcode processing bias. As such, the performance of REBAR can be assessed by comparing the original and bias-corrected fitness estimates against known values (e.g. obtained using independent low-throughput assays). However, such ground truth fitnesses will not always be available for validation, such as when applying REBAR to pre-existing data as we do here. In these cases, the performance of REBAR can be evaluated by comparing the agreement of fitness estimates obtained from replicate assays before and after bias correction.

For example, in the original data, fitness estimates are inconsistent from one replicate to the next for several conditions, including CC1, CC2, KC1, and KC2 (deviation of points from 45-degree line in Fig. 4a). By contrast, the corrections applied by REBAR yield fitness estimates that have high correspondence across replicates (Fig. 4b, CC1). The algorithm had no knowledge of which, if any, assays were expected to be similar, so obtaining high agreement between fitness estimates in known replicate conditions confirms that REBAR is returning accurate fitness corrections. In addition, inferred variant bias susceptibilities are strongly correlated with disagreements between replicate fitness estimates before, but not after, correction (Fig. 4a, CC1), which indicates that REBAR is addressing a bias mode that was responsible for confounding fitness estimates in one or both replicate assays.

Large fitness discrepancies are not observed in the original data for all conditions (e.g. CC3 and KC3 in Fig. 4a). In these cases, REBAR finds negligible bias trends and preserves the original fitness estimates. Therefore, REBAR corrects bias in assays where it distorts fitness estimates while maintaining accuracy where bias is not an issue.

REBAR improves the log-linearity of count trajectories and reduces the overall magnitude of residuals by design (Fig. 4c). Assays with confounding bias prevalence often include count trajectories with large residuals that are substantially reduced by REBAR’s corrections (e.g. CC1 replicate 1 and KC1 replicate 1). However, large residuals are not always associated with misestimates of fitness (e.g. KC3 replicate 1 in Fig. 4c; see also Assay 2 in Fig. 3), and bias-induced errors are not always accompanied by large residuals (e.g. KC2 in Fig. 4c; see also Assay 3 in Fig. 3). The nuanced relationship between bias, residuals, and fitness estimates is difficult to disambiguate on a per-assay basis, but REBAR successfully leverages the global structure of residuals to do so.

In the high-bias conditions, the median magnitude of REBAR’s fitness correction was 0.08 for Kinsler et al. (KC1 and KC2) and 0.02 for Chen et al. (CC1 and CC2). For comparison, the median fitness (after correction) in these two datasets was 0.09 and 0.06, respectively. In other words, the corrections are non-negligible and can be comparable to the magnitude of the fitness effects the assay is meant to measure (for more detailed quantification, see supplementary Section S3, Supplementary Material online).

The variant library assayed in Kinsler et al. (2020) has a special feature that allows us to validate the REBAR-inferred fitness corrections even further. Specifically, this library includes several subsets of variants with similar mutations that are expected to have nearly equal fitnesses (supplementary Section S3.1.2, Supplementary Material online). REBAR has no knowledge of such groups (other than the single required control set). Therefore, demonstrating that fitness estimates within these groups become more similar after the REBAR corrections provides additional independent validation of the method’s accuracy.

In particular, many of the mutants in Kinsler et al. (2020) underwent a whole-genome duplication to become diploid but have otherwise very similar genetic backgrounds. The fitness effect of genome duplication is similar for all diploid mutants across the assays considered here (supplementary Section S3.1.2, Supplementary Material online). Similarly, two other subsets of variants have mutations in the same gene—*GPB2* and *PDE2*, respectively—that confer similar fitness effects across assays (supplementary Section S3.1.2, Supplementary Material online). Any one of these groups can be used as an approximate equal-fitness control set required for our method, and the remaining two groups can then be used for independent validation of the output. Here, we use the diploid set for the inference control, and *GPB2* and *PDE2* for validation.

**Fig. 5.**
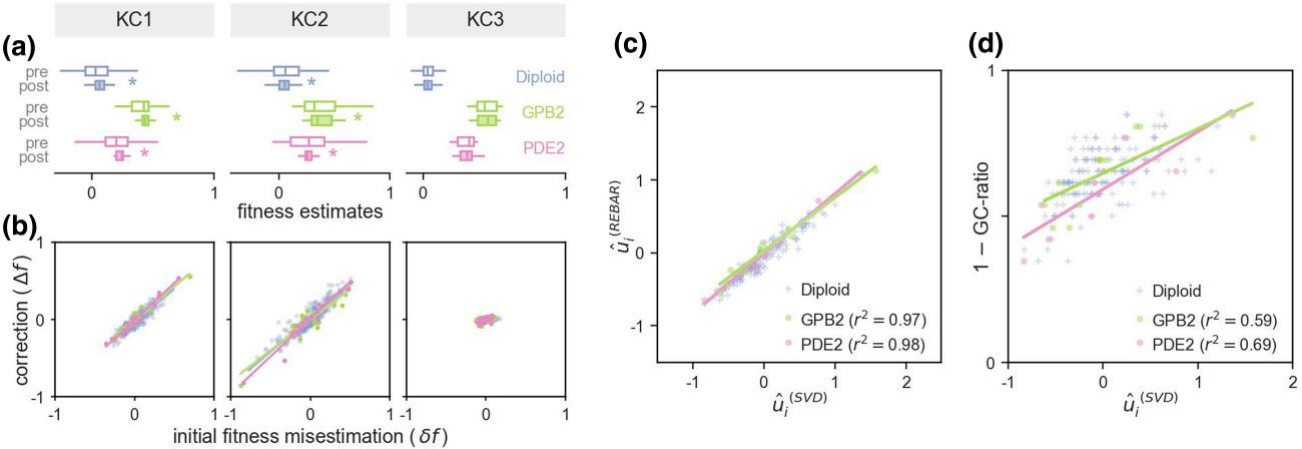
Validation of bias-corrected fitness estimates and inferred bias susceptibilities. a) Distributions of fitness estimates before and after bias correction (white and shaded boxplots, respectively) are shown for the Kinsler et al. (2020) control (diploid) and validation (*GPB2* and *PDE2*) groups of variants across three growth conditions (“Kinsler conditions” KC1, KC2, and KC3). The variance of fitness estimates decreases significantly (statistical significance, denoted by *, is determined by Levene’s test for equal variances; P<0.05) in the KC1 and KC2 conditions, in which multiple assays have high-bias prevalence with strong temporal trends (Fig. 4c). b) The correspondence between each variant’s initial fitness misestimation using pre-correction counts data ($\delta {\;f}_{i\in G}^{\alpha }={\bar{\;f}}_{i\in G}^{\alpha }-\langle {\bar{\;f}}_{G}\rangle^{\alpha }$) and the change in its fitness estimate following bias correction ($\Delta {\;f}_{i\in G}^{\alpha }={\tilde{\;f}}_{i\in G}^{\alpha }-{\bar{\;f}}_{i\in G}^{\alpha }$) is depicted using scatter plots for each growth condition (each point represents a variant). c) The bias susceptibility values returned by REBAR are highly correlated with bias susceptibilities inferred using an independent, SVD-based method, which we consider to be approximate ground truth values. d) As expected, the approximate ground truth bias susceptibility of each variant is also correlated with the GC ratio of its barcode, but this correlation is weaker than it is for the bias susceptibility values returned by REBAR.

The results of this analysis are shown in Fig. 5a. REBAR significantly reduces the variance of fitness estimates for GPB2 and PDE2 mutants in assay conditions where within-group variation was initially high (e.g. KC1 and KC2). We see that significant reductions in fitness estimate variance coincide with assays where bias is both relatively prevalent and has a strong temporal trend (see supplementary Supplementary Figs. S7 and S11, Supplementary Material online). In conditions where fitness estimates are already tight in the original data (e.g. KC3), REBAR independently infers low bias prevalence and does not significantly alter either the mean or variance of fitness estimates. We emphasize that the method is ignorant of the *GPB2* and *PDE2* groupings and thus has no explicit incentive to favor tight distributions of fitness estimates for these subsets of variants. The fact that REBAR does return tighter estimates of fitness for groups that are expected to have near-equal fitness provides validation of its performance.

Figure 5b provides a closer look at how REBAR’s bias corrections impact fitness estimates at the level of individual variants. In conditions where initial fitness estimates have substantial errors (e.g. KC1 and KC2), the updates to fitness estimates induced by our bias-corrections ($\Delta {\;f}_{i\in G}^{\alpha }={\tilde{\;f}}_{i\in G}^{\alpha }-{\bar{\;f}}_{i\in G}^{\alpha }$) are highly correlated with the initial errors in estimation ($\delta {\;f}_{i\in G}^{\alpha }={\bar{\;f}}_{i\in G}^{\alpha }-\langle {\bar{\;f}}_{G}\rangle^{\alpha }$). This demonstrates that the corrections made by REBAR address the underlying cause of misestimation for each particular variant. On the other hand, REBAR does not significantly alter fitness estimates in conditions where fitness errors are already low (e.g. KC3).

Finally, we can also compare the bias components returned by REBAR to those obtained using an independent method. The relationship given by Equation (3) (i.e. $\delta f_{i}^{\alpha }=u_{i}\lambda^{\alpha }$) indicates that if fitness misestimates $\delta f_{i}^{\alpha }$ were known, then bias susceptibilities and prevalence trends could be recovered by performing singular value decomposition (SVD) on a variant-by-assay matrix of fitness errors (specifically, the first left singular vector provides estimates of bias susceptibility for each variant, and the first right singular vector gives estimates of the trend in bias prevalence in each assay; see supplementary Section S3.1.2, Supplementary Material online). We stress that this SVD approach requires knowledge of true fitnesses for the collection of variants and assays in question, which is, of course, not generally available beforehand. However, this SVD can be applied individually to known subsets of equal-fitness variants, such as our *GPB2* and *PDE2* validation groups, for which true fitnesses can be approximated using the subset’s average fitness estimate. This provides yet another avenue for validating the method’s performance.

Figure 5c shows a tight correspondence between the variant bias susceptibility values inferred using REBAR and the corresponding values estimated using the SVD approach. The strong correlation seen in each validation group confirms the bias inference performed by our algorithm. Critically, note that REBAR infers bias susceptibility values for the entire variant library, while the SVD approach can only be applied to special variant subsets for which a priori fitness information is available.

Variants with the strongest inferred susceptibilities are often those with the most extreme GC ratios, as predicted by the structure of residuals in the original data (supplementary Section S3, Supplementary Material online). GC content is known to play a role in modulating how a barcode responds to bias-inducing conditions in the amplification and sequencing process, but it is not the only factor that influences a barcode’s representation in a sample (Kuo et al. 2002; Aird et al. 2011). We find that a barcode’s susceptibility to bias is only weakly correlated with sequence properties such as GC ratio (Fig. 5d) or nucleotide entropy (supplementary Figs. S8 and S12, Supplementary Material online), which indicates that the bias mode corrected by REBAR is poorly explained by simple sequence proxies alone. While we do not characterize all of the factors contributing to the observed barcode processing bias, the bias susceptibility values inferred by REBAR capture each barcode’s response to this multi-factorial bias mode and account for the systematic structure in the data. In turn, the estimates of bias susceptibilities and bias prevalences returned by REBAR may help pinpoint the features of barcodes or protocols that are responsible for bias.

## Discussion

We have developed a robust computational method for inferring and correcting the effects of barcode processing biases in high-throughput fitness assays. REBAR infers the bias susceptibility of each barcode as well as the prevalence of bias in each sample. These components are used to estimate the effect of bias on each individual count, from which bias-adjusted effective counts are obtained. The bias-corrected counts are better representations of the actual changes in variant abundances, and thus provide more accurate estimates of fitness.

An advantage of our approach is that REBAR requires only one multiply labeled subset of variants to infer and correct the effects of bias for an entire library. The accuracy of REBAR depends in part on the fidelity of this control set. Ideally, this group would consist of differentially labeled but otherwise genetically identical strains to ensure absolute fitness neutrality among the control set. When such an ideal control set is not available (such as when analyzing a pre-existing data set), a subset of variants that are believed to be nearly neutral can be used, as was done here for the Chen et al. (2023) and Kinsler et al. (2020) data sets. These case studies demonstrate that REBAR can make significant improvements to fitness estimates even when there is some biological variation in the control set.

**Fig. 6.**
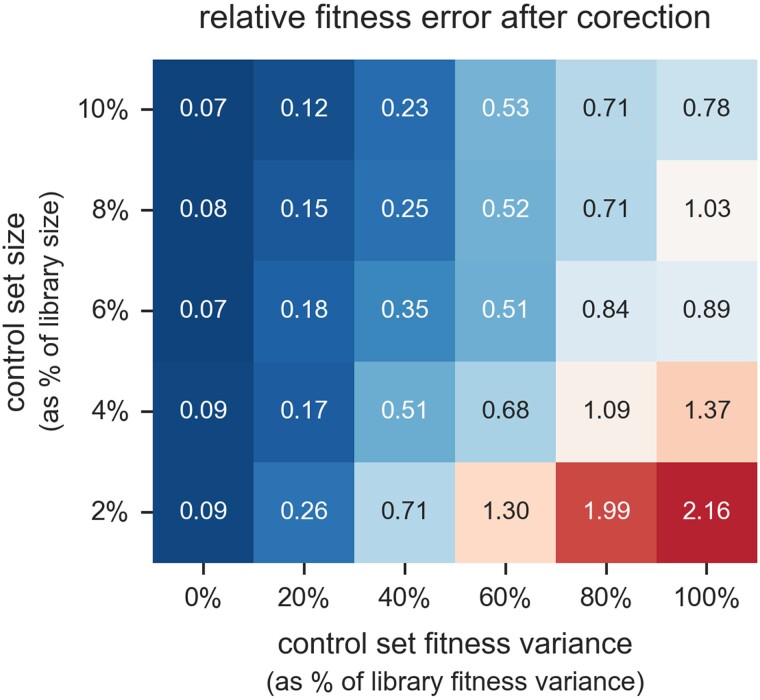
Sensitivity of fitness error reduction to control set fidelity. We tested REBAR on synthetic data sets with simulated noise and bias effects. Each synthetic data set includes barcode read counts for a library of 500 variants from 5 simulated bulk fitness assays, where ground truth fitnesses, bias susceptibilities, and bias prevalences are assigned randomly (see supplementary Section S3.3.2, Supplementary Material online, for details). We measure the mean absolute error (MAE) in fitness estimates for each data set before and after correction by REBAR (${\text{MAE}}_{\mathrm{pre}}$ and ${\text{MAE}}_{\mathrm{post}}$, respectively). We also estimate the error in fitness estimates that is expected of Poisson sampling noise alone in the absence of bias for each data set ${\text{MAE}}_{\mathrm{noise}}$. Each cell in the heatmap reports the median “relative fitness error after correction” ε for 50 synthetic data sets, where $\varepsilon =\left({\text{MAE}}_{\mathrm{post}}-{\text{MAE}}_{\mathrm{noise}}\right)/\left({\text{MAE}}_{\mathrm{pre}}-{\text{MAE}}_{\mathrm{noise}}\right)$. REBAR removes nearly all of the initial fitness error attributable to bias when used with an ideal zero-variance control set (e.g. genetically identical strains). Decreasing control set quality reduces REBAR’s ability to correct fitness estimates, but this can be offset by increasing the size of the control set.

To further evaluate the robustness of REBAR to variation in the control set, we conducted a sensitivity analysis using synthetic data sets with simulated noise and bias effects (details in supplementary Section S3.3.1, Supplementary Material online). We varied the size of the control set and the variance in fitness among control set variants, ranging from identical sets with zero variance to degenerate sets that have the same amount of variance as the library overall (Fig. 6). When the control set has very low fitness variance as intended, REBAR can remove 90% or more of the bias-induced fitness error in these data sets (Fig. 6). The ability to disambiguate bias trends from fitness effects tends to diminish as variation in the control set increases, but substantial error reduction can still be achieved with moderately variable control sets. The accuracy of bias inference improves as the number of control set labels increases, but reasonable accuracy can be achieved with a modest number of nearly neutral control variants. Altogether, we find that REBAR’s ability to significantly reduce bias-induced fitness errors is robust across a wide range of realistic control set sizes and fitness distributions (Fig. 6).

Similarly, the accuracy of bias corrections using this method scales with the size of the data set. In principle, REBAR can make substantial improvements to fitness estimates for even a single assay, but its inference is better constrained and more accurate when applied to multi-assay data sets (supplementary Section S3.3.3, Supplementary Material online). It does not matter if the assays represent replicates from the same condition or assays performed in different conditions so long as the barcoded library is the same throughout. The degree to which inference is improved by the inclusion of additional assays depends on how prevalent bias is in the respective assays.

Here we model the effects of barcode processing bias as a single error mode (i.e. a single coherent pattern of effects). We find that one bias mode is sufficient to correct most of the deviations in the data sets considered here (supplementary Section S3.1.2, Supplementary Material online), but this error model presents two limitations in general. First, REBAR accounts for bias with a “rank-one” structure, but this may not capture all of the systematic sources of error. For example, some structure is still present in our case study residuals following correction by REBAR (supplementary Sections S3.1.3 and S3.2.3, Supplementary Material online), which suggests that there are additional sources of systematic noise beyond the dominant mode that REBAR identifies and removes. In principle, our methodology could be extended to handle multiple bias modes, but we do not investigate that here. Second, REBAR corrects systematic bias but does not address sources of random noise in the growth assay or amplification process. For example, a barcode may become over-represented in a sample due to a one-off PCR jackpot, but this kind of non-systematic effect is outside the scope of REBAR. Unique molecular identifiers (UMIs) are commonly used to detect stochastic variation in PCR amplification (Fu et al. 2011; Kivioja et al. 2012; Johnson et al. 2023), and incorporating UMI information into a REBAR-like error correction pipeline is an area for future work.

A significant benefit of REBAR is that it does not make demands on variant library design or fitness assay protocols, so long as at least one equal-fitness control set is included. This avails REBAR as a post-processing tool that can improve the accuracy of fitness estimates with low overhead in many contexts. Therefore, we anticipate that this computational approach will be applicable to a wide range of experimental fields where accurate fitness measurements are of interest, such as experimental evolution, lineage tracking, and deep mutational scanning.

## Supplementary Material

msae152_Supplementary_Data

## Data Availability

A python module implementing this method is available along with documentation and case study data at github.com/ryansmcgee/REBAR. This implementation is also published as a PyPI package at https://pypi.org/project/rebar-py (installable using, e.g., pip install rebar-py).
