# Supplementary material for: Improving the Accuracy of Bulk Fitness Assays by Correcting Barcode Processing Biases
Source: Mol Biol Evol. 2024 Jul 23;41(8):msae152. doi: 10.1093/molbev/msae152 (PMC11316221; doi:10.1093/molbev/msae152)
Supplement: msae152_Supplementary_Data [file msae152_supplementary_data.pdf]

Supplementary Information

Contents

S1 Bias Model Details & Notation . . . 2
S1.1 Barcode read counts and systematic barcode processing bias . . . 2
S1.2 Linear models of log-counts: Fitness estimates and residuals . . . 3
S1.3 Misestimation of fitness before bias correction . . . 4
S1.4 Bias-corrected counts . . . 5
S1.5 Bracket notations . . . 7
S2 Extended Methods: Bias Inference & Correction Algorithm . . . 8
S2.0 Initialization . . . 8
S2.0.1 Filtering variants with untrustworthy counts . . . 8
S2.0.2 Assigning weights to data points . . . 9
S2.0.3 Initializing bias components . . . 10
S2.1 Inference Stage 1: Inferring bias susceptibilities and bias prevalence 'deviations' . . . 11
S2.1a Inferring variant bias susceptibilities . . . 12
S2.1b Inferring sample bias prevalence deviations . . . 12
S2.2 Inference Stage 2: Inferring bias prevalence trends . . . 14
S2.3 Outputting bias-corrected counts and fitness estimates . . . 16
S2.4 Code availability . . . 16
S3 Case Studies . . . 17
S3.1 Kinsler et al. (2020) Case Study . . . 17
S3.1.1 Fitness assay data . . . 17
S3.1.2 Control set . . . 18
S3.1.3 Supplementary Results . . . 20
S3.2 Chen et al. (2023) Case Study . . . 26
S3.2.1 Fitness assay data . . . 26
S3.2.2 Control set . . . 26
S3.2.3 Supplementary Results . . . 28
S3.3 Sensitivity Analyses . . . 33

S3.3.1 Generation of Synthetic Data 33
S3.3.2 Sensitivity to Control Set 35
S3.3.3 Sensitivity to Number of Assays 35
S3.4 REBAR parameterization 37

S1 Bias Model Details & Notation

S1.1 Barcode read counts and systematic barcode processing bias

We consider data sets collected from bulk fitness assays. These data consist of barcode read count time series collected for a library of variants (indexed by i) from one or more assays (indexed by α; representing e.g., replicates or alternative environmental conditions). The relative count of a barcode among all counts for a given sample is expected to represent the relative abundance of the corresponding variant in the assay culture. We refer to the set of counts supplied to our algorithm as *raw* counts (although these input counts may have been pre-processed for other reasons). The raw read count of barcode (variant) i at time point (sample) t in assay α is denoted by $\mathcal{C}^{\alpha}_{i,t}$.

Several factors can cause raw counts to deviate from relative abundances. First, sequencing depth (i.e., total reads per sample) typically varies from one sample to the next. This can cause counts to exhibit fluctuations that are not representative of changes in the composition of the population. Therefore, counts must be normalized to account for sequencing depth variation within each assay before time series analyses (e.g., growth rates estimation) can be done. This can be achieved by dividing the counts in each sample (t) by a set of per-sample normalization factors $\{Z^{\alpha}_t\}$ that offset differences in total counts across time points:

$$C^{\alpha}_{i,t} = \frac{\mathcal{C}^{\alpha}_{i,t}}{Z^{\alpha}_t} \,. \tag{S6}$$

We refer to the resulting counts $C^{\alpha}_{i,t}$ as *normalized* counts or simply *counts* (distinguishing them from raw input counts and the bias-adjusted counts discussed below). Our method infers appropriate depth-normalization factors as part of the bias inference algorithm (Supplementary Section S2.1b).

Random noise and systematic biases can also cause individual barcode read counts to deviate from accurate representations of relative abundances. Let the "*true*" count of barcode i in sample t of assay

α be given by $\mathbb{C}^{\alpha}_{i,t}$; that is, the count value that would be observed in the absence of barcode processing bias or noise. The corresponding normalized count that is actually observed, $C^{\alpha}_{i,t}$, incorporates the "true" barcode representation as well as the contributions of processing bias $b^{\alpha}_{i,t}$ and noise $\xi^{\alpha}_{i,t}$ on this data point (focusing on log counts here):

$$\log C^{\alpha}_{i,t} = \log \mathbb{C}^{\alpha}_{i,t} + b^{\alpha}_{i,t} + \xi^{\alpha}_{i,t}\,. \tag{S7}$$

Our model of systematic barcode processing bias posits that the bias can be decomposed into two components: the characteristic susceptibility of barcode i to bias, u_i, and the prevalence of bias-inducing effects in sample t of assay α, v^{α}_t:

$$b^{\alpha}_{i,t} = u_i v^{\alpha}_t\,. \tag{S8}$$

Changes in bias prevalence over time can confound estimates of growth rates. To address temporal trends in bias prevalence, we describe the prevalence of bias at time (sample) t of assay α using a linear model:

$$v^{\alpha}_t = \lambda^{\alpha} t + v^{\alpha}_0 + \gamma^{\alpha}_t\,, \tag{S9}$$

where the slope λ^{α} gives the overall trend in bias over the course of the assay, and γ^{α}_t values represent the deviations from this trend for each individual sample (and where v^{α}_0 is the y-intercept).

S1.2 Linear models of log-counts: Fitness estimates and residuals

We assume that bulk fitness assays are conducted using conditions where the variants of interest segregate exponentially (e.g., variants of interest make up a small fraction of the population relative to the reference strain), or that the raw data has been pre-processed to account for deviations from this assumption. Under this assumption, the log-count of barcode i is expected to change linearly with a slope that corresponds to the fitness (growth rate) of the associated variant. Therefore, an estimate of fitness $\bar{f}^{\alpha}_i$ can be obtained by fitting a linear model to the corresponding normalized counts:

$$\log \bar{C}^{\alpha}_{i,t} = \bar{f}^{\alpha}_i t + \bar{c}_i^{\alpha}\,, \tag{S10}$$

where $\bar{c}_i^{\,\alpha}$ is the y-intercept and $\log \bar{C}_{i,t}^{\alpha}$ is the predicted log-count of barcode i in sample t under the best-fit linear model (the 'bar' notation denotes quantities inferred from the *observed* counts data). An observed log-count deviates from the corresponding best-fit line by the residual amount $\bar{r}_{i,t}^{\,\alpha}$:

$$\log C_{i,t}^{\alpha} = \log \bar{C}_{i,t}^{\alpha} + \bar{r}_{i,t}^{\,\alpha}\,. \tag{S11}$$

S1.3 Misestimation of fitness before bias correction

Given that observed counts incorporate the effects of bias and noise, estimates of fitnesses given by the slopes of lines fit to observed count trajectories may deviate from the true fitnesses of variants. Suppose that we had access to "true" counts that were unaffected by bias or noise. In the absence of these deviation effects, true counts would follow perfectly log-linear trajectories with slopes that represent the true fitness $f_{i,\mathrm{true}}^{\alpha}$ of each variant (where c_i^{α} is the y-intercept of this trajectory):

$$\log \mathbb{C}_{i,t}^{\alpha} = f_{i,\mathrm{true}}^{\alpha} t + c_i^{\alpha}\,. \tag{S12}$$

The amount by which the fitness estimate obtained from observed data deviates from the true fitness is given by

$$\delta f_i^{\alpha} = \bar{f}_i^{\alpha} - f_{i,\mathrm{true}}^{\alpha}\,. \tag{S13}$$

This misestimation in fitness is equal to the difference between the 'rise' of the observed and true count trajectories over the 'run' of the time series (here we only care about the difference in slopes, so we can assume that the two count trajectories are y-shifted to have the same y-intercepts $c_i^{\alpha} = \bar{c}_i^{\,\alpha}$ w.l.o.g.):

$$\begin{aligned}
\delta f_i^{\alpha} &= \underbrace{\frac{1}{\tau}}_{\text{run}} \left[\Big(\underbrace{\log \bar{C}_{i,\tau}^{\alpha} - \bar{c}_i^{\,\alpha}}_{\text{rise of observed}}\Big) - \Big(\underbrace{\log \mathbb{C}_{i,\tau}^{\alpha} - c_i^{\alpha}}_{\text{rise of true}}\Big)\right] && \text{using Eq. S10 \& Eq. S12, time series of length } \tau \\
&= \frac{1}{\tau}\left(\log \bar{C}_{i,\tau}^{\alpha} - \log \mathbb{C}_{i,\tau}^{\alpha}\right) && \text{where } c_i^{\alpha} = \bar{c}_i^{\,\alpha}
\end{aligned}$$

We can evaluate this difference further:

$$\begin{aligned}
\delta f_i^\alpha &= \frac{1}{\tau}\left(\left(\log C_{i,\tau}^\alpha - \bar{r}_{i,\tau}^\alpha\right) - \log \mathbb{C}_{i,\tau}^\alpha\right) && \text{using Eq. S11} \\
&= \frac{1}{\tau}\left(\left(\log \mathbb{C}_{i,\tau}^\alpha + b_{i,\tau}^\alpha + \xi_{i,\tau}^\alpha - \bar{r}_{i,\tau}^\alpha\right) - \log \mathbb{C}_{i,\tau}^\alpha\right) && \text{using Eq. S7} \\
&= \frac{1}{\tau}\left(b_{i,\tau}^\alpha + \xi_{i,\tau}^\alpha - \bar{r}_{i,\tau}^\alpha\right) \\
&= \frac{1}{\tau}\left(u_i v_\tau^\alpha + \xi_{i,\tau}^\alpha - \bar{r}_{i,\tau}^\alpha\right) && \text{using Eq. S8} \\
&= \frac{1}{\tau}\left(u_i\left(\lambda^\alpha \tau + \gamma_\tau\right) + \xi_{i,\tau}^\alpha - \bar{r}_{i,\tau}^\alpha\right) && \text{using Eq. S9, let } v_0^\alpha = 0 \text{ w.l.o.g.} \\
&= u_i \lambda^\alpha + \frac{1}{\tau}\left(\left(u_i \gamma_\tau + \xi_{i,\tau}^\alpha\right) - \bar{r}_{i,\tau}^\alpha\right)
\end{aligned}$$

The first term tells us that the error in fitness estimated from observed counts follows from the assay's trend in bias prevalence, as mediated by the degree of bias susceptibility for the variant in question. The second term is a contribution to this error related to the difference between the observed residual for the final sample and the incidental effects of bias and noise on that sample. This difference can be interpreted as frustration in the linear fit that is *not* accounted for by the effects of bias or noise in barcode processing (i.e., the mapping of true counts to observed counts, Eq. S7), such as deviations from exponential (log-linear) trajectories of abundances in the assay itself. The contribution of the second term is expected to be small and diminishes as the length of the time series increases, such that the error in fitness estimation will be dominated by the first term. That is, the misestimation of the fitness of variant i is equal (to first order) to the trend in bias prevalence as mediated by that variant's bias susceptibility:

$$\delta f_i^\alpha = u_i \lambda^\alpha \,. \tag{S14}$$

S1.4 Bias-corrected counts

We improve the accuracy of fitness estimates by calculating a set of adjusted counts that 'remove' the effect of bias from the values that are used to fit a log-linear model. That is, the bias-adjusted count for barcode i in sample t of assay α, denoted by $A_{i,t}^\alpha$, is obtained by subtracting the estimated bias effect

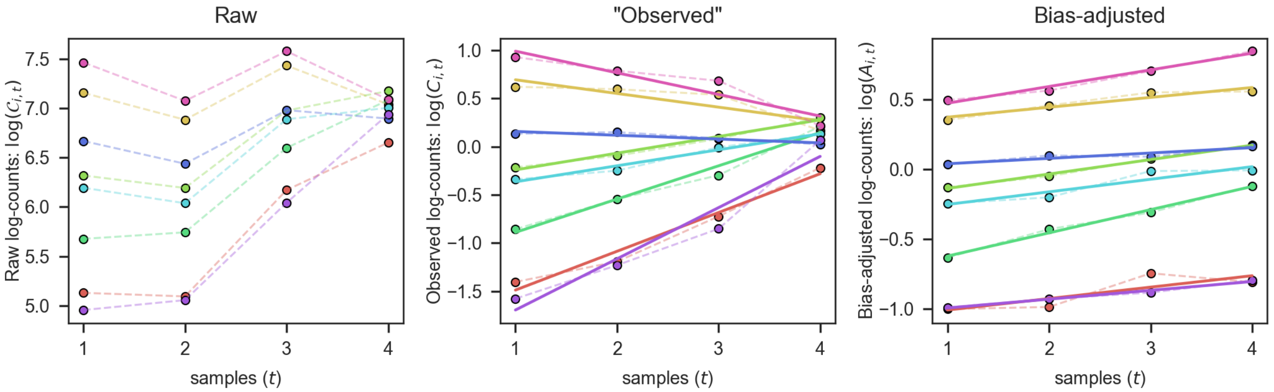

Figure S1: **Example of updates to counts data using our method. (Left)** Raw log-count trajectories (dashed lines) for eight variants (different colors) in the 'EC2 rep2' assay from the Kinsler et al. (2020) data set are shown (see Supplementary Section S3 for more information). The depicted variants are all members of the near-equal fitness control set used in our case study (see Supplementary Section S3.1.2), so they are expected to have log-count trajectories with nearly-equal slopes. **(Center)** "Observed" normalized log-counts trajectories obtained by normalizing counts to account for varying sequencing depth (dashed lines; Eq. S6) and the corresponding best fit linear regressions (solid lines; Eq. S10) are shown. The depth-normalization makes observed log-count trajectories more linear than the raw trajectories. However, there is large variance in the slopes of the observed log-linear trajectories, which is unexpected for near-equal fitness variants and indicative of systematic bias. **(Right)** Log-count trajectories following bias-adjustment using our method (dashed lines; Eq. S15) and the corresponding best fit linear regressions (solid lines; Eq. S17) are shown. The bias-correction reduces the magnitude of residuals on average and results in fitness estimates that are more accurate, as seen in a decrease in the variance of slopes (i.e., fitness estimates) for this set of near-equal fitness variants.

$\hat{b}^{\alpha}_{i,t}$ from the observed count for that barcode and sample:

$$\log A^{\alpha}_{i,t} = \log C^{\alpha}_{i,t} - \hat{b}^{\alpha}_{i,t}\,. \tag{S15}$$

Following our model of bias (Eq. S8), we decompose the estimated bias into estimates for the underlying bias susceptibility and prevalence components:

$$\log A^{\alpha}_{i,t} = \log C^{\alpha}_{i,t} - \hat{u}_i \hat{v}^{\alpha}_t \tag{S16}$$

Accurate estimates of the bias components (i.e., $u_i v^{\alpha}_t \approx \hat{u}_i \hat{v}^{\alpha}_t$) will bring the bias-adjusted counts $A^{\alpha}_{i,t}$ closer to the true counts $\mathbb{C}^{\alpha}_{i,t}$, especially when random noise is negligible:

$$\log A^{\alpha}_{i,t} = \log \mathbb{C}^{\alpha}_{i,t} + u_i v^{\alpha}_t + \xi^{\alpha}_{i,t} - \hat{u}_i \hat{v}^{\alpha}_t \qquad \text{using Eq. S16, Eq. S7, \& Eq. S8}$$

$$\log A^{\alpha}_{i,t} - \log \mathbb{C}^{\alpha}_{i,t} = u_i v^{\alpha}_t - \hat{u}_i \hat{v}^{\alpha}_t \qquad \text{where } \xi^{\alpha}_{i,t} \approx 0$$

Fitting a linear model to the adjusted counts yields a bias-adjusted estimate of fitness:

$$\log \widetilde{A}_{i,t}^{\alpha} = \widetilde{f}_i^{\alpha} t + \widetilde{c}_i^{\alpha} \,, \tag{S17}$$

where $\widetilde{c}_i$ is the y-intercept and $\log \widetilde{A}_{i,t}^{\alpha}$ is the predicted adjusted log-count of barcode i in sample t under the best-fit linear model (the 'tilde' notation denotes quantities inferred from the *adjusted* counts data). An adjusted log-count ($\log A_{i,t}^{\alpha}$) deviates from the corresponding best-fit line by the residual $\widetilde{r}_{i,t}^{\alpha}$:

$$\log A_{i,t}^{\alpha} = \log \widetilde{A}_{i,t}^{\alpha} + \widetilde{r}_{i,t}^{\alpha} \,. \tag{S18}$$

Note that the values of bias-adjusted counts and their corresponding residuals depend on the bias susceptibility and bias prevalence values that are used to compute the adjustment.

S1.5 Bracket notations

- **Sets:** Curly brackets denote a set of values. A set contains all elements corresponding to the indices written inside the brackets, while the set is associated with a specific element of the index written outside the brackets (if any). For example, $\{\widetilde{r}_{i,t}\}^{\alpha}$ denotes the set of residuals for all variants and all time points associated with a particular assay α, whereas $\{\widetilde{r}_t^{\alpha}\}_i$ denotes the set of residuals from all assays and all time points for a particular variant i.

- **Averages:** Angle brackets denote the arithmetic mean of a set of values, where the ensemble that is averaged includes all elements corresponding to the indices written inside the brackets, and indices written outside the brackets specify elements that are fixed with respect to the average (consistent with the set notation above). For example, $\langle C_i \rangle_t^{\alpha}$ denotes the average count of all variants for a particular sample t from a particular assay α (i.e., $\langle C_i \rangle_t^{\alpha} = \frac{1}{N} \sum_{i=1}^{N} C_{i,t}^{\alpha}$).

S2 Extended Methods: Bias Inference & Correction Algorithm

Our bias inference and correction method proceeds through the following phases:

S2.0 Initialization

S2.0.1 Filtering variants with untrustworthy counts

Variants that have very low or very high abundances in a given assay may have read counts that are impacted by factors other than barcode processing bias, such as high counting error, zero counts in some samples, or deviations from exponential growth (log-linear trajectories). Variant i is considered to have '*trustworthy*' data in assay α if its average raw count across samples in that assay falls between designated minimum and maximum mean-count thresholds (i.e., $\theta_{\min} \leq \langle C_t \rangle_i^\alpha \leq \theta_{\max}$; threshold values used in our case studies are given in Supplementary Section S3.4). To avoid extreme counts unduly influencing the inference of bias components, we exclude data for 'untrustworthy' variants from certain parts of our procedure, as described below.

- **Inference of bias prevalence deviations:** Bias prevalence deviation values are inferred on a per-assay basis, using the set of residuals over variants and samples for each assay (i.e., $\{\widetilde{r}_{i,t}\}^\alpha$; see Supplementary Section S2.1b). Variants that are not trustworthy in a particular assay are excluded from the set of residuals used to infer bias prevalence deviations for that assay (although they may be included in other assays where they are trustworthy).

- **Inference of bias susceptibility:** Bias susceptibility values are inferred on a per-variant basis, using the set of residuals from all assays and samples for each variant (i.e., $\{\widetilde{r}_t^{\,\alpha}\}_i$; see Supplementary Section S2.1a). If a variant does not have trustworthy data in at least Φ assays, then its bias susceptibility is not inferred but rather fixed at zero (the value of Φ used in our case studies is given in Supplementary Section S3.4).

- **Inference of bias prevalence trends:** Bias prevalence trend values are inferred on a per-assay basis, using inferred bias susceptibility values and estimated fitness errors for a pre-designated control set of equal-fitness variants (Supplementary Section S2.2). In order to be included in the trend inference for a particular assay, a control-set variant must have trustworthy data for that assay and have met the trustworthiness criteria for bias susceptibility inference (i.e., meeting both

criteria outlined above).

S2.0.2 Assigning weights to data points

We infer bias components and depth-normalization factors using variations of least squares regression, which infers the parameter values that generate adjusted counts with the smallest sum of squared residuals (relative to the best-fit log-linear model; see Supplementary Section S2.1 for details). However, not all counts, and therefore not all residuals, are equally reliable. For example, the counts of low-abundance variants are expected to be more impacted by counting noise (i.e., have a greater variance-to-mean ratio) than those of high-abundance variants. To account for variability in count precision, we use forms of Weighted Least Squares (WLS) regression, in which each residual $\widetilde{r}_{i,t}^{\alpha}$ is given a weight $w_{i,t}^{\alpha}$ that determines its relative 'importance' in the regression.

In WLS regression, it is standard to assign weights equal to the reciprocal of the variance of the observations. Here the relevant observations are log-counts, for which we must determine the variance. Let the raw read count of variant i in sample t of assay α be a Poisson-distributed random variable $\chi_{i,t}^{\alpha} \sim \mathrm{Pois}(k_{i,t}^{\alpha})$. The mean and variance of this count distribution (i.e., $k_{i,t}^{\alpha}$) depends on the culture abundance of this variant in this sample, but we do not assume that we have this information. Instead, we receive a raw count measurement $C_{i,t}^{\alpha}$. Based on a single measurement, the best estimate of the mean and variance of $\chi_{i,t}^{\alpha}$ is $C_{i,t}^{\alpha}$ itself. However, our regressions take place in log-count space, so we still need to determine $\mathrm{Var}(\log(\chi_{i,t}^{\alpha}))$. Finding the variance of a non-linear transformation $g(X)$ of a random variable X is non-trivial, but the delta method provides an approximation when the variance of X is known:

$$\mathrm{Var}(g(X)) \approx \left(g'(\mathrm{E}[X])\right)^2 \mathrm{Var}(X)\,. \tag{S19}$$

In our case, we have $g(X) = \log(X)$, $g'(X) = 1/X$, $\mathrm{E}[\chi_{i,t}^{\alpha}] \approx C_{i,t}^{\alpha}$, and $\mathrm{Var}(\chi_{i,t}^{\alpha}) \approx C_{it}^{\alpha}$. Plugging these

into the delta method equation above, we have:

$$\mathrm{Var}(\log(\chi_{i,t}^{\alpha})) \approx \left(\frac{1}{\mathrm{E}[\chi_{i,t}^{\alpha}]}\right)^2 \mathrm{Var}(\chi_{i,t}^{\alpha}) \tag{S20}$$

$$= \left(\frac{1}{\mathcal{C}_{i,t}^{\alpha}}\right)^2 \mathcal{C}_{i,t}^{\alpha} \tag{S21}$$

$$= \frac{1}{\mathcal{C}_{i,t}^{\alpha}}\,. \tag{S22}$$

Therefore, the variance of the log-count of variant i in sample t of assay α is well-approximated by $1/\mathcal{C}_{i,t}^{\alpha}$.

Defining the weight of a data point as the reciprocal of the corresponding observation's variance, we have:

$$w_{i,t}^{\alpha} = \frac{1}{\mathrm{Var}(\log(\chi_{i,t}^{\alpha}))} = \frac{1}{\frac{1}{\mathcal{C}_{i,t}^{\alpha}}} = \mathcal{C}_{i,t}^{\alpha}\,. \tag{S23}$$

We modify this weight assignment slightly, capping the maximum weight at a designated maximum weight threshold, W_{max}:

$$w_{i,t}^{\alpha} = \min\left(\mathcal{C}_{i,t}^{\alpha},\ W_{\mathrm{max}}\right)\,. \tag{S24}$$

This reflects an assumption that all sufficiently large count observations are equally reliable for our inference purposes.

S2.0.3 Initializing bias components

Our method infers sets of depth-normalization, bias susceptibility, and bias prevalence values using non-linear least squares optimization, as described in the following sections. Initial values for each of these sets must be specified to serve as initial conditions to the optimization algorithm. Our package allows the user to provide initial values for these sets, but default initializations are presented here:

- **Initial depth-normalization factors:** By default, initial normalization factor values are set to the mean raw counts for the respective samples (i.e., $\acute{Z}_{t,\mathrm{init}}^{\alpha} = \langle C_i \rangle_t^{\alpha}$). *Note: Depth-normalization factors must not be zero.*

- **Initial bias susceptibilities:** By default, initial bias susceptibility values are drawn from a

normal distribution with zero mean and small variance: $\{\acute{u}_{i,\text{init}}\} \sim \mathcal{N}(\mu_u, \sigma_u)$ (values used in our case studies are given in Supplementary Section S3.4).

- **Initial bias prevalences:** By default, initial bias prevalence values are drawn from a normal distribution with zero mean and small variance: $\{\acute{v}^{\alpha}_{t,\text{init}}\} \sim \mathcal{N}(\mu_v, \sigma_v)$ (values used in our case studies are given in Supplementary Section S3.4).

Note: Bias susceptibilities and bias prevalences should not be initialized to zero, as this can cause the gradient of the bias effect ($\acute{b}^{\alpha}_{i,t} = \acute{u}_i \acute{v}^{\alpha}_t$) to be zero, which impairs convergence of the optimization.

S2.1 Inference Stage 1: Inferring bias susceptibilities and bias prevalence 'deviations'

This stage of the method infers the depth-normalization factors, variant bias susceptibilities, and bias prevalence deviation terms that jointly optimize the log-linearity (i.e., minimize the set of residuals) of the correspondingly-adjusted count trajectories. Recall that the estimated effect of bias on an observed count value $C^{\alpha}_{i,t}$ is modeled by the product of bias suceptibility and bias prevalence components ($\hat{b}^{\alpha}_{i,t} = \hat{u}_i \hat{v}^{\alpha}_t$, Eq. S8). Subtracting the estimated bias effects from all observed counts yields a set of bias-adjusted counts (Eq. S16), and fitting a linear model to the adjusted counts yields a set of "bias-adjusted residuals" $\{\widetilde{r}_{i,t}\}^{\alpha}$ (Eq. S17, Eq. S18). As such, the bias-adjusted residual values that are obtained depend on the bias component values that are used (as well as the normalization factors used to produce the observed counts):

$$\{C_{i,t}\}^{\alpha}, \{\hat{Z}_t\}^{\alpha} \xrightarrow[\text{Eq. S6}]{\text{normalization}} \{C_{i,t}\}^{\alpha} \xrightarrow[\text{Eq. S16}]{\text{bias-adjustment}} \{A_{i,t}\}^{\alpha} \xrightarrow[\text{Eq. S17, Eq. S18}]{\text{log-linear fit}} \widetilde{f}^{\alpha}_i, \{\widetilde{r}_{i,t}\}^{\alpha}$$

$$\hat{u}_i, \{\hat{v}_t\}^{\alpha} \;\text{(input to bias-adjustment)}$$

Figure S2: Dependence of bias-adjusted residuals on bias components and depth-normalization factors.

At this stage, we iterate over the following optimization steps (described below) until suitable convergence of the inferred values is reached (as determined by a numerical precision threshold or designated number of iterations).

- **Stage 1a**: Inferring bias susceptibilities
- **Stage 1b**: Inferring bias prevalence deviations
- *Bias component re-normalization step*

S2.1a Inferring variant bias susceptibilities

For each variant (with adequate trustworthy data; see Supplementary Section S2.0.1), we infer the bias susceptibility value that minimizes that variant's consequent bias-adjusted residuals across all samples and assays. In particular, we use numerical non-linear least squares optimization to solve for the susceptibility value that satisfies:

$$\hat{u}_i = \underset{\acute{u}_i}{\operatorname{argmin}} \left[\sum_{\alpha,t} w_{i,t}^{\alpha} \left(\widetilde{r}_{i,t}^{\,\alpha} \right)^2 + \eta_u \left(\acute{u}_i \right)^2 \right] , \tag{S25}$$

where all bias prevalences $\{\acute{v}_t\}^{\alpha}$ and normalization factors $\{\acute{Z}_t\}^{\alpha}$ are fixed at their present values. This optimization is a weighted least squares regression with a ridge-like penalty η_u on the magnitude of the susceptibility value. Weighting the regression gives counts with more reliable counts more influence over the susceptibility inference (for more information about weights $w_{i,t}^{\alpha}$ see Supplementary Section S2.0.2). The penalty term favors small susceptibility values and limits outliers.

S2.1b Inferring sample bias prevalence deviations

For each assay, we infer a set of per-sample bias prevalence values that minimize the consequent collection of residuals over all time points and variants (with trustworthy data for the given assay; see Supplementary Section S2.0.1). In particular, we use numerical non-linear least squares optimization to solve for the bias prevalence values that satisfy:

$$\{\hat{v}_t\}^{\alpha}, \{\hat{Z}_{t_2,\ldots,t_{\tau-1}}\}^{\alpha} = \underset{\{\acute{v}_t\}^{\alpha},\, \{\acute{Z}_{t_2,\ldots,t_{\tau-1}}\}^{\alpha}}{\operatorname{argmin}} \left[\sum_{i,t} w_{i,t}^{\alpha} \left(\widetilde{r}_{i,t}^{\,\alpha} \right)^2 + \eta_v \sum_{t} \left(\acute{v}_t^{\alpha} \right)^2 \right] , \tag{S26}$$

where all bias susceptibilities $\{\hat{u}_i\}$ are fixed at their present values. This optimization is a weighted least squares regression with a ridge-like penalty η_v on the magnitude of the prevalence values. Weighting the regression gives counts with more reliable counts more influence over the inference (for more information about weights $w_{i,t}^{\alpha}$ see Supplementary Section S2.0.2).

In this step, the penalty term plays two roles. First, it regularizes the inference, which favors small prevalence values and limits over-fitting. Second, it is important to note that the log-count residuals ($\{\widetilde{r}_{i,t}\}^{\alpha}$) reflect the bias prevalence deviations ($\{\hat{\gamma}_{i,t}\}^{\alpha}$) from an underlying linear trend in bias prevalence ($\hat{\lambda}^{\alpha}$)(Eq. S9). As a result, an infinite number of different bias prevalence time series with different slopes

but the same deviations will result in the same residuals. This 'zero mode' (symmetry) must be resolved in order for the optimization to converge and for the resulting bias prevalence values to not include arbitrary trends. The penalty term resolves this by favoring small bias prevalence values that in turn bias the inference to sequences of prevalences with near-zero slope ($\hat{\lambda}^{\alpha} \approx 0$).

In this stage of the method, we implicitly enforce that there are no temporal trends in bias prevalence, and therefore inferring bias prevalence values is equivalent to inferring the bias prevalence *deviations* from the near-zero trend (i.e., $\hat{\lambda}^{\alpha} \approx 0 \;\Rightarrow\; \{\hat{v}_t\}^{\alpha} \approx \{\hat{\gamma}_t\}^{\alpha}$; Eq. S9). In Stage 2 of the method, we relax this assumption and infer the actual trend in bias prevalence for each assay, using the inferred trend and deviation values to update the absolute bias prevalence estimates for all samples.

Notice that this step optimizes depth-normalization factors jointly with bias prevalence inference. More precisely, we fix the normalization factors for the first and last time points of the assay (t_1 and t_τ, respectivley), and we optimize the remaining intermediate time points ($t_2, \ldots, t_{\tau-1}$) along with the bias prevalence values. Fixing the first and last time points prevents the sequence of normalization factors from drifting arbitrarily and impairing convergence due to the interaction of normalization factors and bias prevalence values in determining adjusted counts and consequent residuals.

Bias component re-normalization step We model the effect of bias on a given log-count as the product of bias susceptibility and bias prevalence components (Eq. S8). However, this product is only defined up to a common factor a:

$$\hat{b}^{\alpha}_{i,t} = \hat{u}_i \hat{v}^{\alpha}_t = (a\,\hat{u}_i)\left(\frac{\hat{v}^{\alpha}_t}{a}\right)$$

That is, an infininte number of combinations of $\hat{u}_i$, $\hat{v}^{\alpha}_t$, and a values can give the same bias effect value $\hat{b}^{\alpha}_{i,t}$. This must be resolved in order for our iterative inference process to converge. We do so by fixing the factor a to a particular value and re-normalizing bias component values accordingly during each iteration of Inference Stage 1. In particular, we do the following:

1. Rescale the current bias susceptibilities: Multiply all bias susceptibility values by a common factor a, which is defined such that this multiplication rescales the collection of all bias susceptibilities to have a fixed target standard deviation σ^*_u:

$$\hat{u}_i = a\,\hat{u}_i\,,$$

where

$$a = \frac{\sigma_u^*}{\mathrm{stdev}(\{\hat{u}_i\})}\,.$$

2. Re-normalize the current bias prevalences: Divide all bias prevalence values by the common factor a defined above, which re-normalizes the prevalence values to the newly rescaled susceptibility values and maintains the absolute bias effects:

$$\hat{v}_t^\alpha = \frac{\hat{v}_t^\alpha}{a}\,.$$

S2.2 Inference Stage 2: Inferring bias prevalence trends

Coming out of Stage 1, we have obtained a set of inferred bias susceptibility values $\{\hat{u}_i\}$ for all variants and sets of bias prevalence values $\{\hat{v}_t\}^\alpha$ for all assays. In Stage 1, our regularization enforced that inferred bias prevalence values have negligible temporal trends (i.e., near-zero slopes) within each assay, so these values are better interpreted as sets of approximate bias prevalence deviation values $\{\hat{\gamma}_t\}^\alpha$ ($\lambda^\alpha \approx 0 \;\Rightarrow\; \{\hat{v}_t\}^\alpha \approx \{\hat{\gamma}_t\}^\alpha$). In Stage 2, we infer the actual trend in bias prevalence for each assay, using the inferred trend and deviation values to determine the absolute bias prevalence for all samples.

To infer bias prevalence trends, we make use of the relationship between fitness misestimates (before bias correction) and bias susceptibility (Eq. S14):

$$\delta f_i^\alpha = u_i \lambda^\alpha\,.$$

This relationship tells us that the trend in bias prevalence for an assay can be estimated by regressing misestimates of fitness from that assay against bias susceptibilities for a set of variants for which these values are known.

In general, fitness misestimate information is not available in the context of fitness assays. However, our method requires that the assays under consideration include a designated control set of equal-fitness variants, which we denote as $G := \{i \mid f_{i,\mathrm{true}}^\alpha = f_{G,\mathrm{true}}^\alpha\}$. That is, the variants in the control set G are assumed to share the same 'true' fitness, $f_{G,\mathrm{true}}^\alpha$. The true fitness of the control variants can be estimated

by taking the average of the observed fitness estimates $\bar{f}_i$ among the control variants:

$$f^{\alpha}_{G,\text{true}} \approx \langle \bar{f}_{i\in G}\rangle^{\alpha}\,. \tag{S27}$$

Then the misestimate of fitness for each variant in the control set can be estimated by

$$\begin{aligned} \delta f^{\alpha}_{i\in G} &= \bar{f}_{i\in G} - f^{\alpha}_{G,\text{true}} && \text{(S28)}\\ &\approx \bar{f}_{i\in G} - \langle \bar{f}_{i\in G}\rangle^{\alpha}\,. && \text{(S29)} \end{aligned}$$

Therefore, inclusion of a control set of equal-fitness variants avails a set of variants for which both fitness misestimates and bias susceptibilities can be obtained.

We use these values to estimate the trend in bias prevalence for each assay using ordinary least squares regression:

$$\hat{\lambda}^{\alpha} = \underset{\acute{\lambda}^{\alpha}}{\operatorname{argmin}} \left[\sum_{i\in G} \left(\delta f^{\alpha}_{i\in G} - \hat{u}_{i\in G}\acute{\lambda}^{\alpha} \right)^2 \right] \tag{S30}$$

At this point, we have inferred sets of bias prevalence deviation values $\{\hat{\gamma}_t\}^{\alpha}$ (from Stage 1) as well as bias prevlance trend values $\hat{\lambda}^{\alpha}$ (from Stage 2) for each assay. These temporal trends in bias were not resolvable from the residuals-based inference in Stage 1, but we can now compute bias prevalence values that incorporate this temporal information and reflect the actual effects of bias on each assay (Eq. S9):

$$\hat{v}^{\alpha}_t = \hat{\lambda}^{\alpha} t + \hat{\gamma}_t{}^{\alpha}\,. \tag{S31}$$

Since it is the slope of bias prevalences not their absolute value that ultimately impacts fitness estimates, we conclude this stage by centering the bias prevalence values for each assay (such that the y-intercept is near zero) without loss of generality:

$$\hat{v}^{\alpha}_t \Leftarrow: \hat{v}^{\alpha}_t - \langle \hat{v}_t \rangle^{\alpha}\,. \tag{S32}$$

S2.3 Outputting bias-corrected counts and fitness estimates

At the end of Stage 2, we possess a set of inferred bias susceptibility values $\{\hat{u}_i\}$ for all variants, sets of (absolute) bias prevalence values $\{\hat{v}_t\}^\alpha$ for all assays, and inferred depth-normalization factors for all samples $\{\hat{Z}_t\}^\alpha$. We use the final inferred values to compute a final set of bias-adjusted counts for all variants and all assays (Eq. S16). Log-linear models fit to these final bias-adjusted counts yield final bias-adjusted fitness estimates (as well as final bias-adjusted residuals; Eq. S17). The output of our algorithm includes the final bias-corrected counts and fitness estimates, as well as other metadata.

S2.4 Code availability

A python module implementing this method is available along with documentation and case study data at `github.com/ryansmcgee/REBAR`. This implementation is also published as a PyPI packge at `https://pypi.org/project/rebar-py` (installable using, e.g., `pip install rebar-py`).

S3 Case Studies

S3.1 Kinsler et al. (2020) Case Study

S3.1.1 Fitness assay data

We applied REBAR to the yeast bulk fitness assay data from Kinsler et al. (2020). These fitness assays were performed with barcoded mutants (variants) isolated from a previous evolution experiment (Levy et al., 2015), which were competed against a constructed reference strain with a restriction site in the barcode region (Venkataram et al., 2016). Variants are labeled with unique 26-base-pair barcodes. This library was assayed in a total of 45 environments (i.e., abiotic culture conditions). These environments include instances of the limited glucose "evolution condition" (EC) in which the library's variants evolved, as well as a range of environments that each feature a small perturbation to this condition, such as changes in the amount of glucose, carbon, or stressors present (e.g., salt). Refer to Kinsler et al. (2020) for additional information about yeast strains, variant mutations, and assay culture conditions.

In this case study, we restrict our analysis to data from environments for which all corresponding samples have a total sequencing depth of at least 100,000 reads. We analyze data from 35 assays across 15 environmental conditions, as detailed in the table below. For concision, the main text only displays results for three of the EC conditions (referred to as 'Kinsler Conditions' (KC) in the main text), but data from all 35 assays listed below were used in the inference. Results for all conditions can be found in Supplementary Section S3.1.3.

Condition Label	Replicates	Description of culture manipulation
EC21 **(KC1 in main text)**	3	Limited glucose evolution condition
EC20 **(KC2 in main text)**	3	Limited glucose evolution condition
EC23 **(KC3 in main text)**	3	Limited glucose evolution condition
EC18	3	Limited glucose evolution condition
EC13	3	Limited glucose evolution condition
EC3	3	Limited glucose evolution condition
ECBB	4	Limited glucose evolution condition, "EC 1BigBatch" in Kinsler et al. (2020)
Baffled	2	Used baffled flask for incubation
1.4%Gluc	2	Decreased glucose concentration to 1.4%
1.6%Gluc	2	Increased glucose concentration to 1.6%
1.8%Gluc	2	Increased glucose concentration to 1.8%
0.5%Raf	2	Added raffinose at concentration of 0.5%
1.5%Suc1%Raf	1	Omitted glucose, added sucrose at 1.5% and raffinose at 1%
0.2MKCl	1	Added KCl at concentration of 0.2 M
0.5MKCl	1	Added KCl at concentration of 0.5 M

Table S3.1.1 Assay conditions included in this analysis.

S3.1.2 Control set

Our method requires that a subset of variants with near-equal fitnesses be designated for use as a control set (Supplementary Section S2.2). Ideally, the variant library would be designed with a multiply-barcoded but otherwise identical set of strains for this purpose. Here, however, we are applying REBAR to data from fitness assays that were conducted without this consideration in mind, so we must identify a suitable subset of variants with near-equal fitnessses *a posteriori*.

The Kinsler et al. (2020) data set includes 549 variants, each of which represents a mutation that arose in response to the limited glucose evolution conditions. A large number of these mutations fall in a small number of genes, including *GPB2* and *PDE2*. In addition, many variants are diploids resulting from whole-genome duplication events but have otherwise very similar genetic backgrounds. These classes of shared mutation targets offer candidate groups for variants with near-equal fitness effects.

To select groups of putatively near-equal fitness variants, we carried out the following outlier exclusion procedure for each of the aforementioned mutation classes (i.e., *GPB2*, *PDE2*, and Diploids), First, we computed the fitness of each variant using the uncorrected observed counts for each assay (Eq. S10), obtaining a vector of 35 fitness estimates for each variant. We also calculated the vector of mean fitness estimates for each mutation class. We computed the Mahalanobis distance of each variant's fitness vector from its mutation class's mean vector, and variants with distances beyond the 90th quantile of the associated chi-squared distribution were called as outliers and excluded. The remaining variants constitute a group of putatively near-equal fitness variants for the given mutation class.

To gauge the validity of these putative groups, we computed the SVD spectrum of the matrix of fitness misestimates (Eq. S13; variants as rows, assays as columns) for each mutation class (Figure S3). If a group of variants has the same true fitness, then differences in observed fitness estimates among the group are only attributable to systematic bias (assuming the effects of random noise on fitness estimates are small), and the effects of a single bias mode (consistent with our model of bias) result in matrix of fitness misestimates of rank one. Therefore, a matrix of fitness misestimates with a SVD spectrum that is dominated by the first mode is consistent with the corresponding group of variants having nearly equal true fitnesses. We found that the first mode accounted for at least 60% of the variance in fitness misestimates for the Diploid, *GP2*, and *PDE2* groups (Figure S3), which supports the suitability of these variants groups as near-equal fitness sets. In our case study, we used the largest of these groups—the Diploids—as the control set for our method, and we used the *GP2* and *PDE2* groups as validation

groups (see Results, Figure 5).

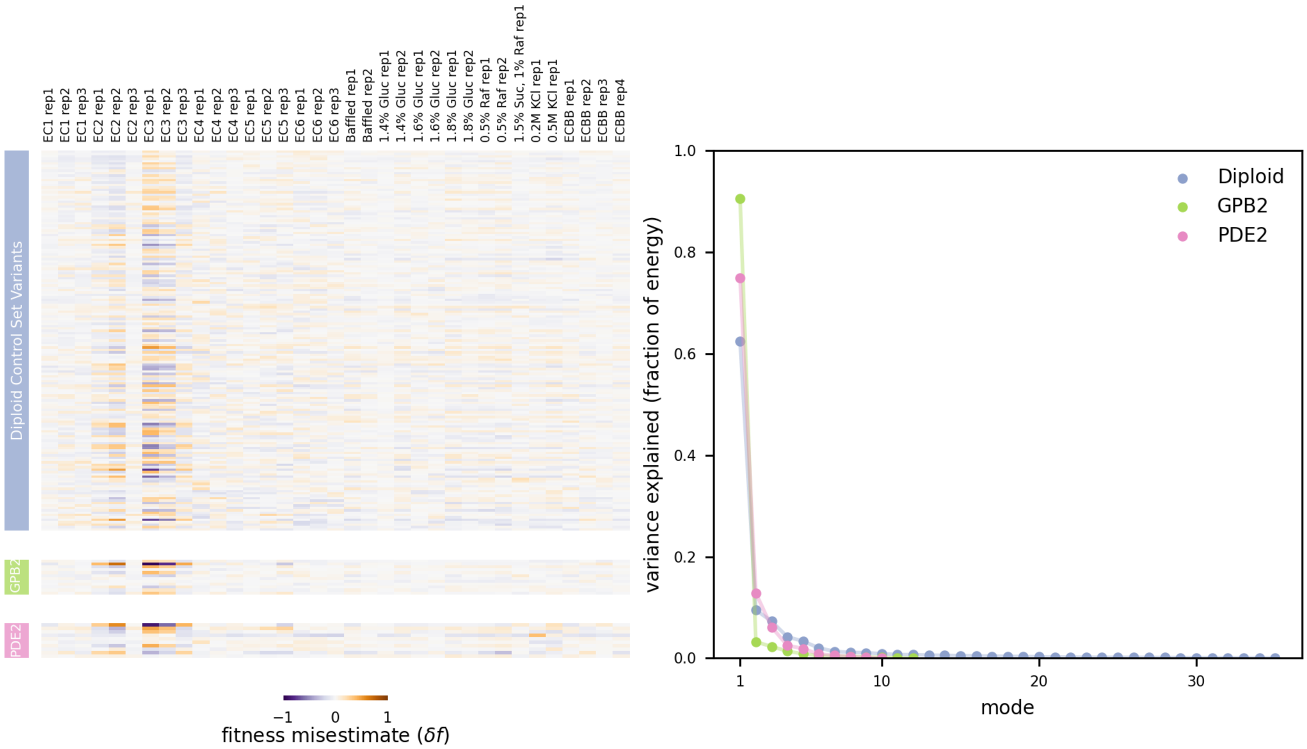

Figure S3: **(Left)** The heatmaps depict the matrix of fitness misestimates for each variant (row) in each assay (column) for the diploid (top, blue), *GPB2* (middle, green), and *PDE2* (bottom, pink) mutation classes. (**Right**) The SVD spectrum for each of the fitness misestimate matrices (shown at left) is plotted. The spectrum for each mutation class is dominated by the first mode, which is consistent with a single bias mode accounting for the observed fitness misestimates.

S3.1.3 Supplementary Results

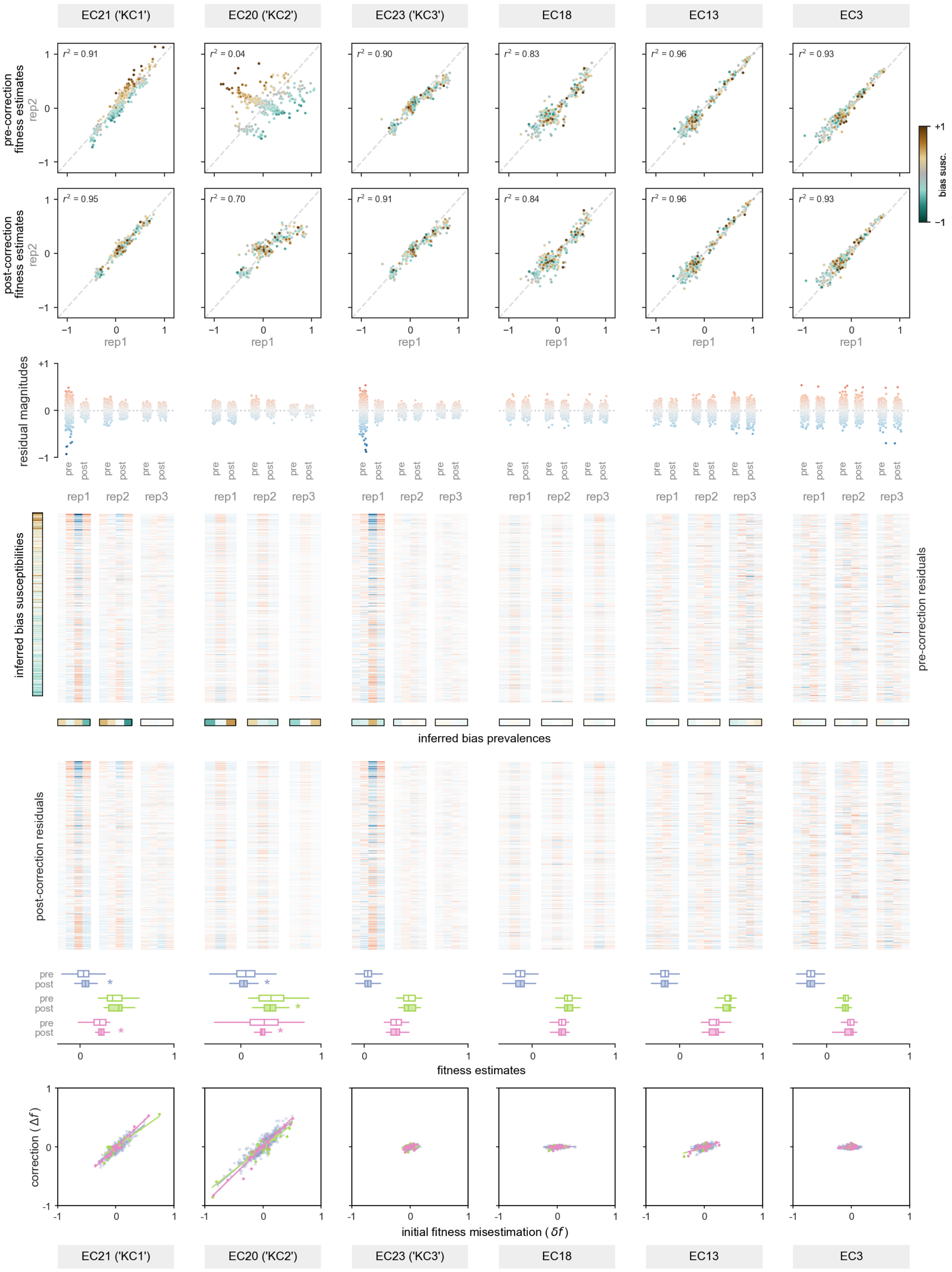

Figure S4: **Results for the Kinsler et al. (2020) case study (Part 1/3).** See Figure S6 for full caption.

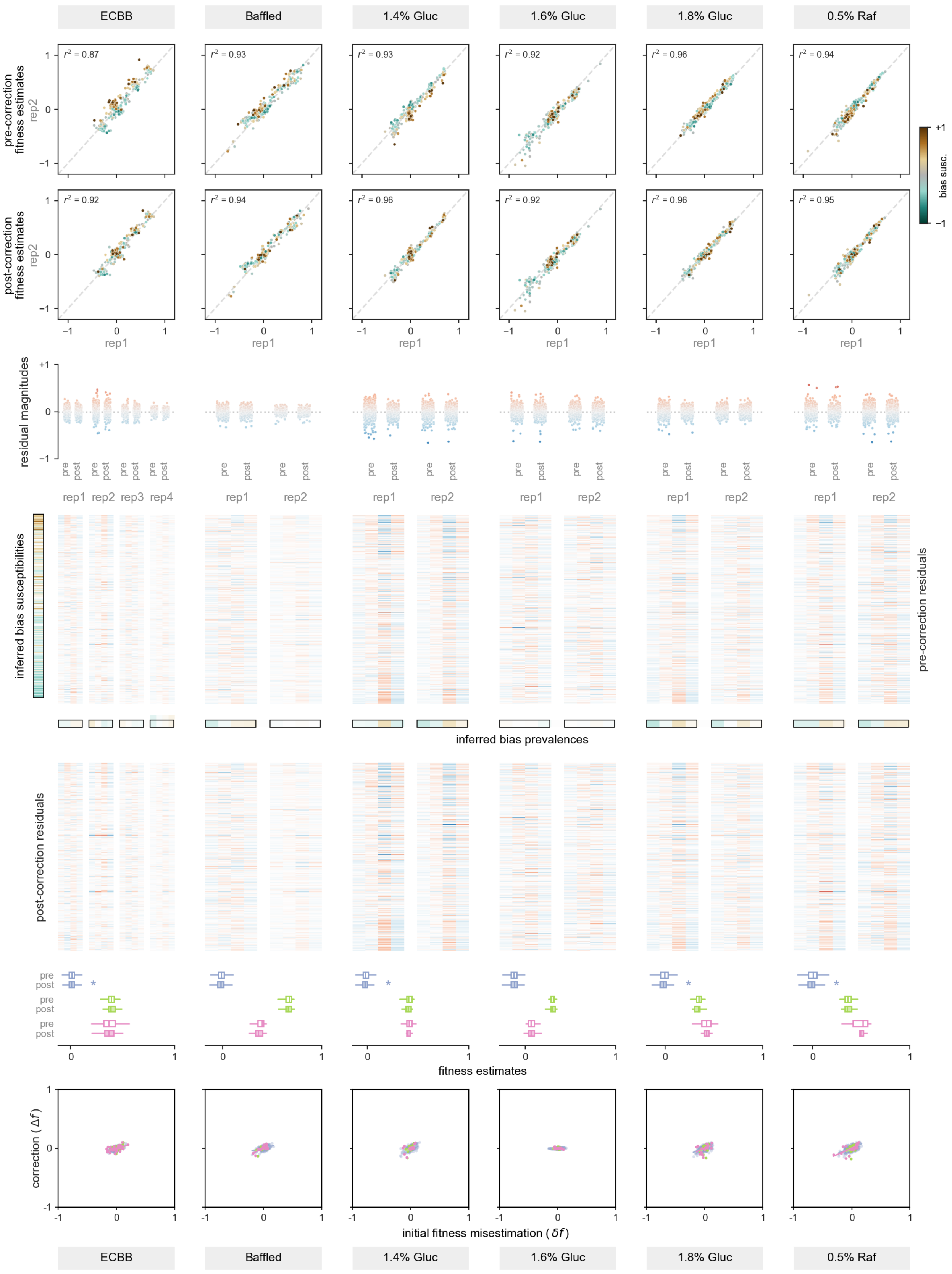

Figure S5: **Results for the Kinsler et al. (2020) case study (Part 2/3)** See Figure S6 for full caption.

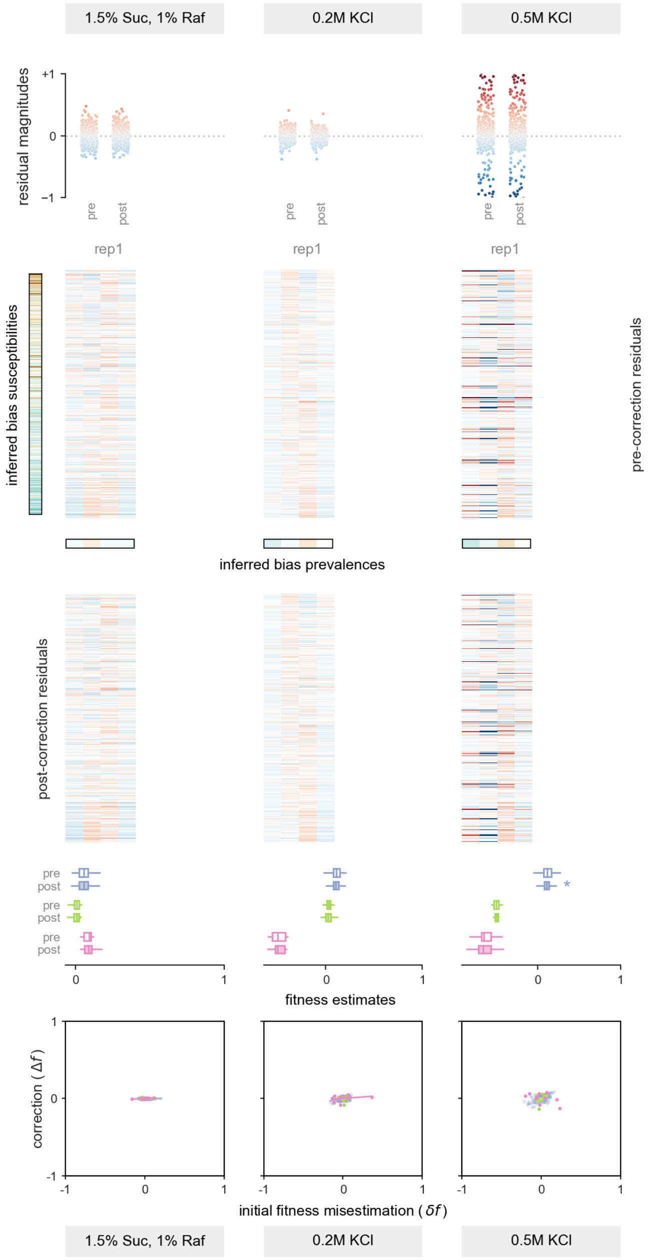

Figure S6: **Results for the Kinsler et al. (2020) case study (Part 3/3)** (Caption also applies to Parts 1 & 2) **(Scatter plots, top; Parts 1 & 2 only)** Fitness estimates from replicate assays in the same environmental conditions are plotted for all variants (points) before and after bias correction. Variants (points) are colored by the bias susceptibility values inferred by REBAR. **(Jitter plots)** Distributions of residual magnitudes for log-linear fits of variant trajectories are shown for pre-correction (raw) counts as well as post-correction (bias-adjusted) counts (each point represents the residual for one variant-sample; distributions combine data from all replicates). **(Heatmaps)** Red-blue heatmaps show the structure of residuals across all variants (rows, sorted by GC ratio increasing top-to-bottom) and samples (columns) before bias correction (above) and after bias correction (below; residuals color scale as in jitter plots). The vertical brown-teal heatmap to the left shows the inferred bias susceptibility value inferred for each variant, and the brown-teal tiles in between the pre- and post-correction residuals heatmaps depict the inferred bias prevalence for the respective samples (bias color scale in upper scatter plots). **(Box plots)** Distributions of fitness estimates before and after bias correction (white and shaded box plots, respectively) are shown for the control set (Diploid) and validation groups (*GPB2* and *PDE2*). Statistically significant reductions in the variance of fitness estimates are denoted by $*$ (Levene's test for equal variances; $p < 0.05$). **(Scatter plots, bottom)** The correspondence between each variant's initial fitness misestimation using pre-correction counts data ($\delta f_i^\alpha = f_{i\in G}^\alpha - \langle f_{j\in G}^\alpha \rangle$) and the change in its fitness estimate following bias correction ($\Delta f_i^\alpha = \widetilde{f}_i^\alpha - f_i^\alpha$) is depicted using scatter plots for each growth condition (each point represents a variant).

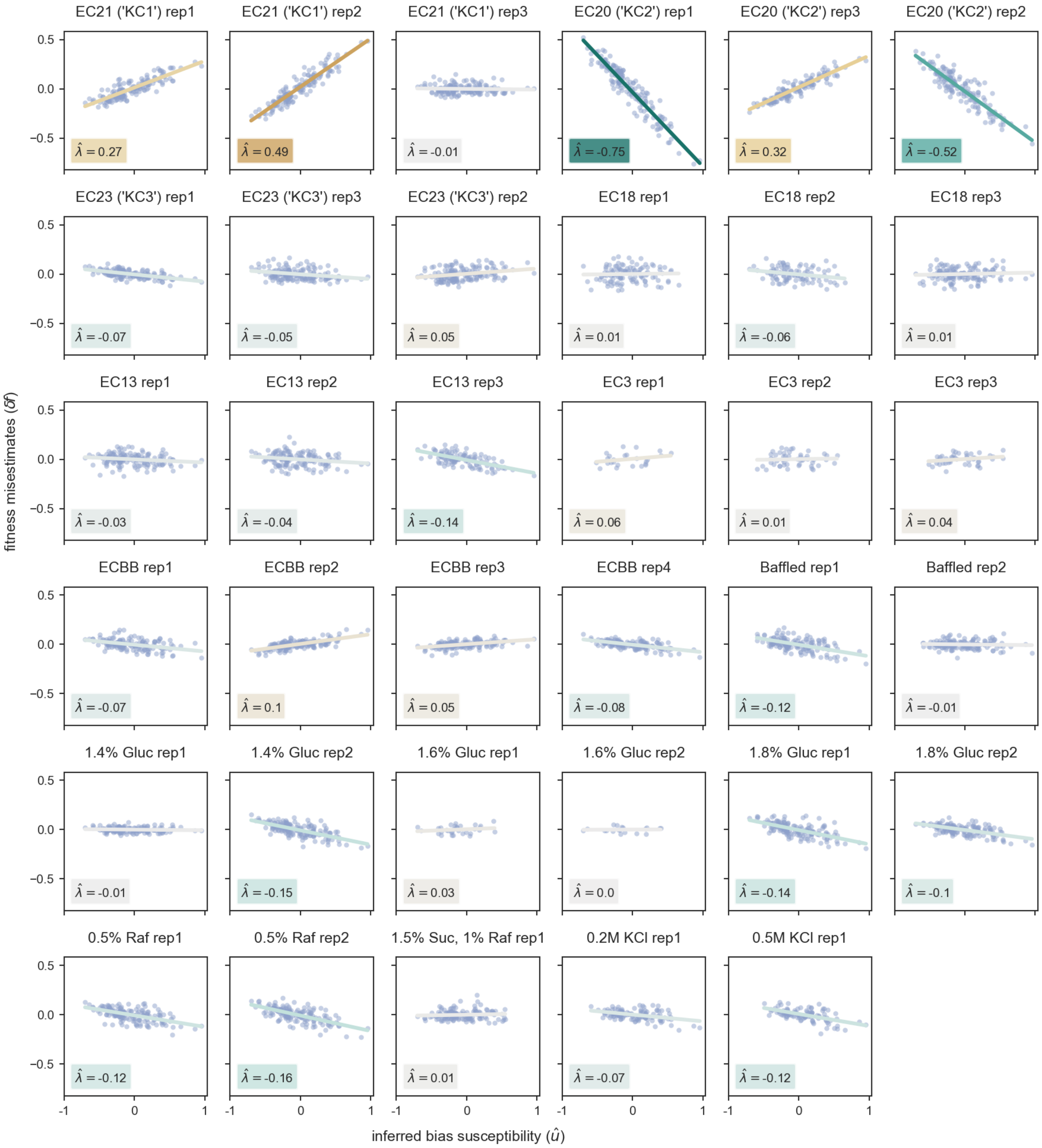

Figure S7: **Inference of bias prevalence trends (Algorithm Stage 2).** The trend in bias prevalence for an assay can be estimated by regressing misestimates of fitness from that assay against bias susceptibilities for a control set of variants for which these values are known (Eq. 3, Supplementary Section S2.2). Each panel presents the results of this regression for one of the 35 assays in the Kinsler et al. (2020) case study data set. Each point represents a variant in the Diploid control set (Supplementary Section S3.1.2). Only control variants that are 'trustworthy' with respect to bias susceptibility inference (Supplementary Section S2.0.1) in a given assay are included in that assay's regression. The best fit line for each assay is shown, the slope of which corresponds to the inferred bias prevalence trend value $\hat{\lambda}^{\alpha}$ for the assay (values listed in the bottom left of each panel).

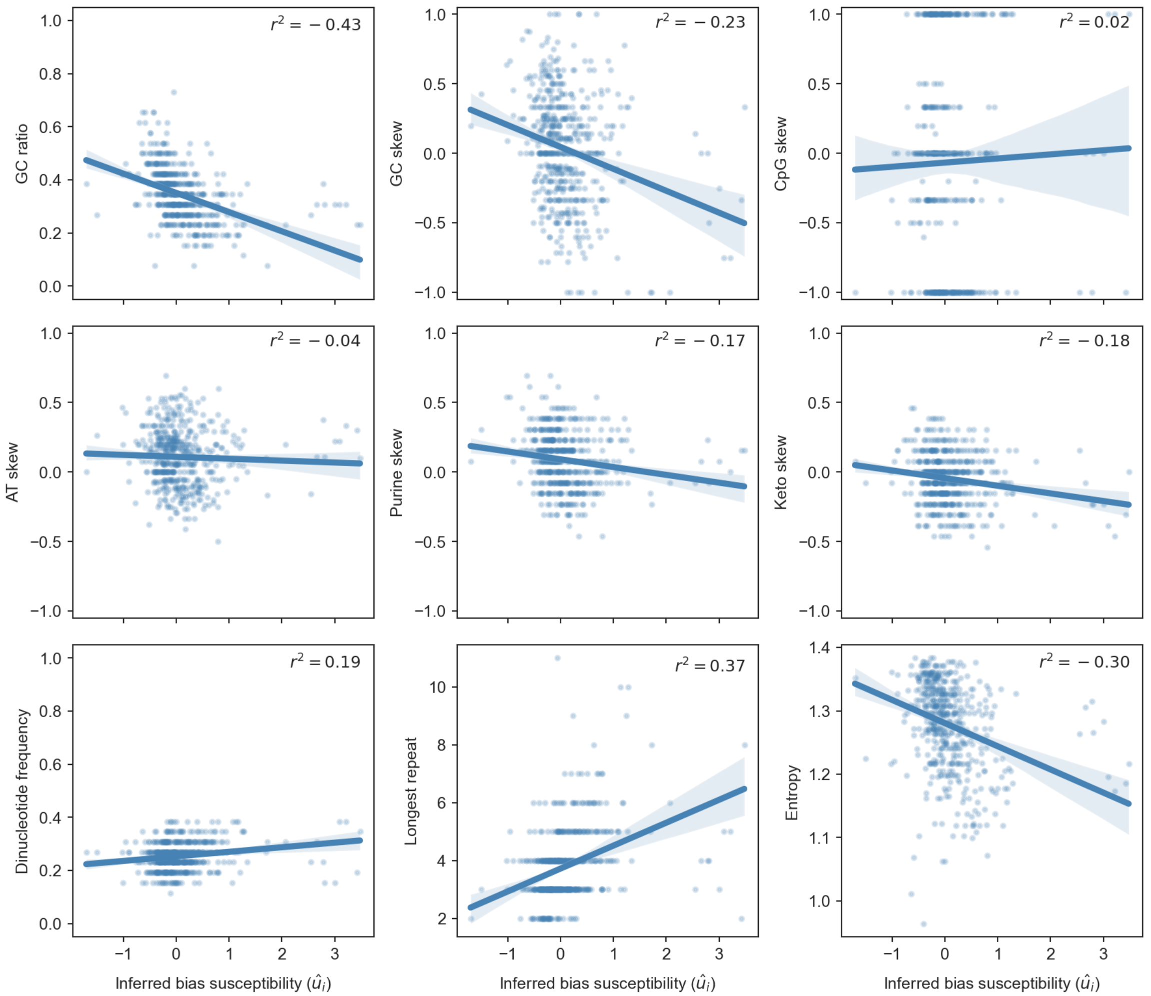

Figure S8: **Correlation of inferred bias susceptibilities with barcode sequence properties.** The bias susceptibility values inferred by REBAR are plotted against various barcode sequence properties for all variants in the Kinsler et al. (2020) library. The best fit line and coefficient of determination (r^2) are shown for each association. While bias susceptibility appears to be weakly correlated with sequence properties, REBAR can capture and correct its multi-factorial effects.

	Median	Mean	RMS	Std Dev
**High bias conditions (KC1, KC2)**				
Pre-correction fitness estimates ($\bar{f}_i^\alpha$)	0.16	0.22	0.29	0.19
Post-correction fitness estimates ($\tilde{f}_i^\alpha$)	0.09	0.20	0.34	0.28
Fitness corrections ($\Delta f_i^\alpha = \tilde{f}_i^\alpha - \bar{f}_i^\alpha$)	0.08	0.14	0.27	0.23
**All conditions**				
Pre-correction fitness estimates ($\bar{f}_i^\alpha$)	0.15	0.20	0.28	0.19
Post-correction fitness estimates ($\tilde{f}_i^\alpha$)	0.13	0.20	0.27	0.19
Fitness corrections ($\Delta f_i^\alpha = \tilde{f}_i^\alpha - \bar{f}_i^\alpha$)	0.01	0.04	0.11	0.10

Table S3.1.3 Statistics of fitness estimates and fitness corrections for trustworthy variants in the Kinsler et al. (2020) data set.

S3.2 Chen et al. (2023) Case Study

S3.2.1 Fitness assay data

We also applied REBAR to bulk fitness assay data from Chen et al. (2023). The authors experimentally evolved barcoded yeast populations in 12 conditions that had a range of environmental or chemical perturbations Chen et al. (2023). Lineages were then isolated from the evolution conditions, genome sequenced to identify mutations, and pooled to form libraries of derived variants. Variants are labeled with two-part barcode system, in which one 26-base-pair sequence encodes the environment in which a variant evolved and an additional 26-base-pair sequence is used for lineage tracking within that environment. We consider the combined 52-base-pair barcode sequence for our purposes. Chen et al. (2023) create three pooled libraries (haploid variants only, haploid and diploid, diploid only), but we focus on the haploid pool here (labeled "hBFA" in Chen et al. (2023)). A bulk fitness assay was conducted to measure the fitnesses of the pooled variants in 9 of the original evolution conditions. Refer to Chen et al. (2023) for additional information about yeast strains, variant mutations, and assay culture conditions.

We analyze data from 17 assays across 9 environmental conditions, as detailed in the table below. For concision, the main text only displays results for three of these conditions (referred to as 'Chen Conditions' (CC) in the main text), but data from all 17 assays listed below were used in the inference. Results for all conditions can be found in Supplementary Section S3.2.3.

Condition Label	Replicates	Description of culture manipulation
37°C **(CC1 in main text)**	2	High temperature
pH3.8 **(CC2 in main text)**	2	Defined rich media buffered to pH 3.8
pH7.3 **(CC3 in main text)**	2	Defined rich media buffered to pH 7.3
SC	2	Defined rich medium
48 hr	2	Defined rich media, diluted every 48 hr
21°C	2	Low temperature
YPD	2	Undefined rich medium, YP + 2% glucose
GlyEtOH	2	Nonfermentable carbon source, 2% glycerol + 2% ethanol
FLC4	1	Antifungal drug, 4 mg/L fluconazole

Table S3.2.1 Assay conditions included in this analysis.

S3.2.2 Control set

Our method requires that a subset of variants with near-equal fitnesses be designated for use as a control set (Supplementary Section S2.2). Ideally, the variant library would be designed with a multiply-barcoded but otherwise identical set of strains for this purpose. Here, however, we are applying REBAR to data

for a library that was created without this consideration in mind, so we must identify a suitable subset of variants with near-equal fitnessses *a posteriori*.

The Chen et al. (2023) data set includes a total of 2,586 variants, each of which represents a mutant lineage that arose in the original study's experimental evolution phase. For each of their pooled libraries, Chen et al. identify a set of 'putatively neutral barcodes' that consists of clones from one evolution environment that all have similar fitness values and show very little adaptation in their lineage tracking data. For the haploid ("hBFA") library that we use here, Chen et al. identify a subset of haploid clones isolated from the YPD evolution condition as their putatively neutral set. We employ the same subset of variants as the control set for the application of REBAR to these data.

S3.2.3 Supplementary Results

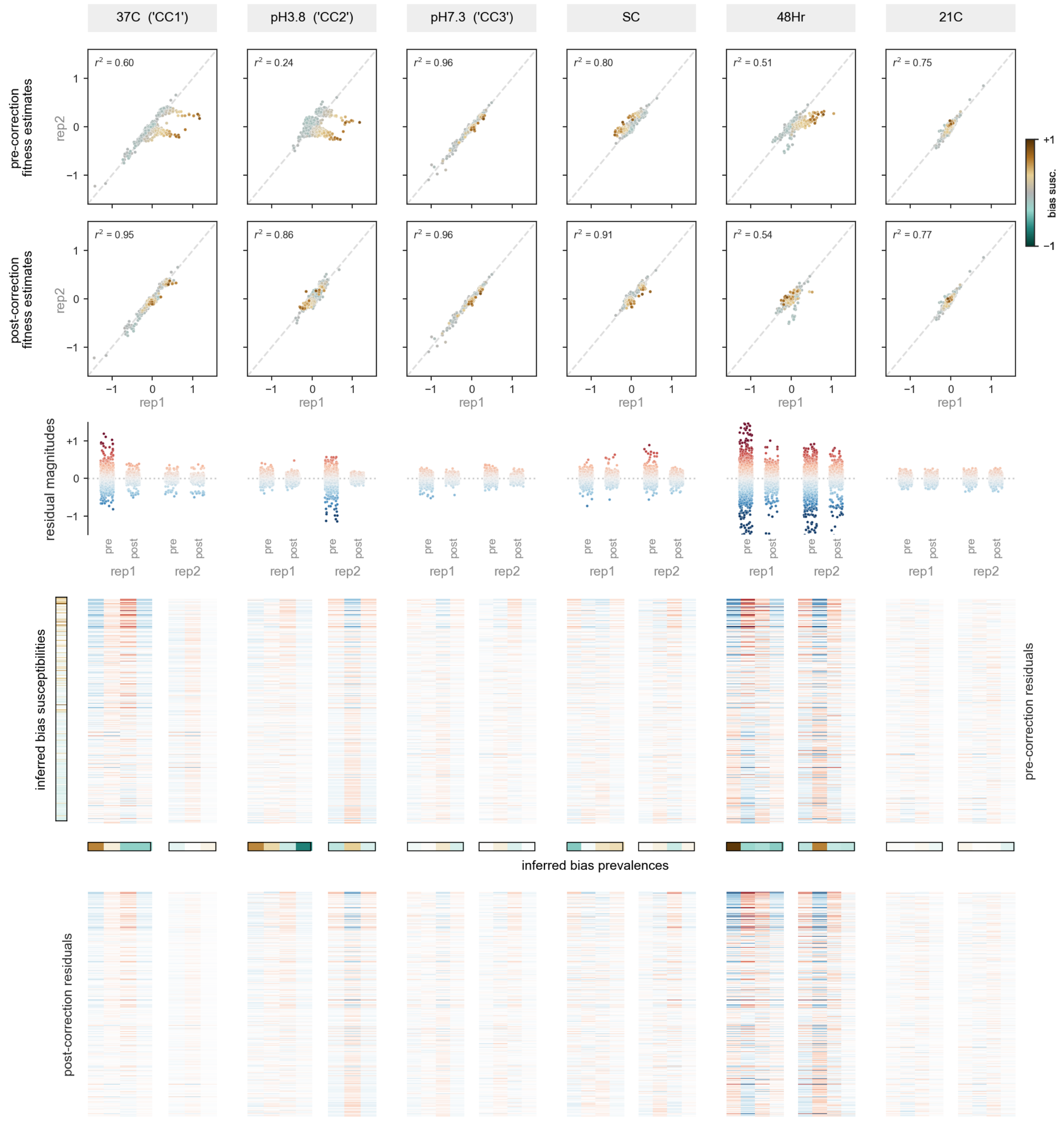

Figure S9: **Results for the Chen et al. (2023) case study (Part 1/2)**
(Scatter plots) Fitness estimates from replicate assays in the same environmental conditions are plotted for all variants (points) before and after bias correction. Variants (points) are colored by the bias susceptibility values inferred by REBAR. **(Jitter plots)** Distributions of residual magnitudes for log-linear fits of variant trajectories are shown for pre-correction (raw) counts as well as post-correction (bias-adjusted) counts (each point represents the residual for one variant-sample; distributions combine data from all replicates). **(Heatmaps)** Red-blue heatmaps show the structure of residuals across all variants (rows, sorted by GC ratio increasing top-to-bottom) and samples (columns) before bias correction (above) and after bias correction (below; residuals color scale as in jitter plots). The vertical brown-teal heatmap to the left shows the inferred bias susceptibility value inferred for each variant, and the brown-teal tiles in between the pre- and post-correction residuals heatmaps depict the inferred bias prevalence for the respective samples (bias color scale in upper scatter plots).

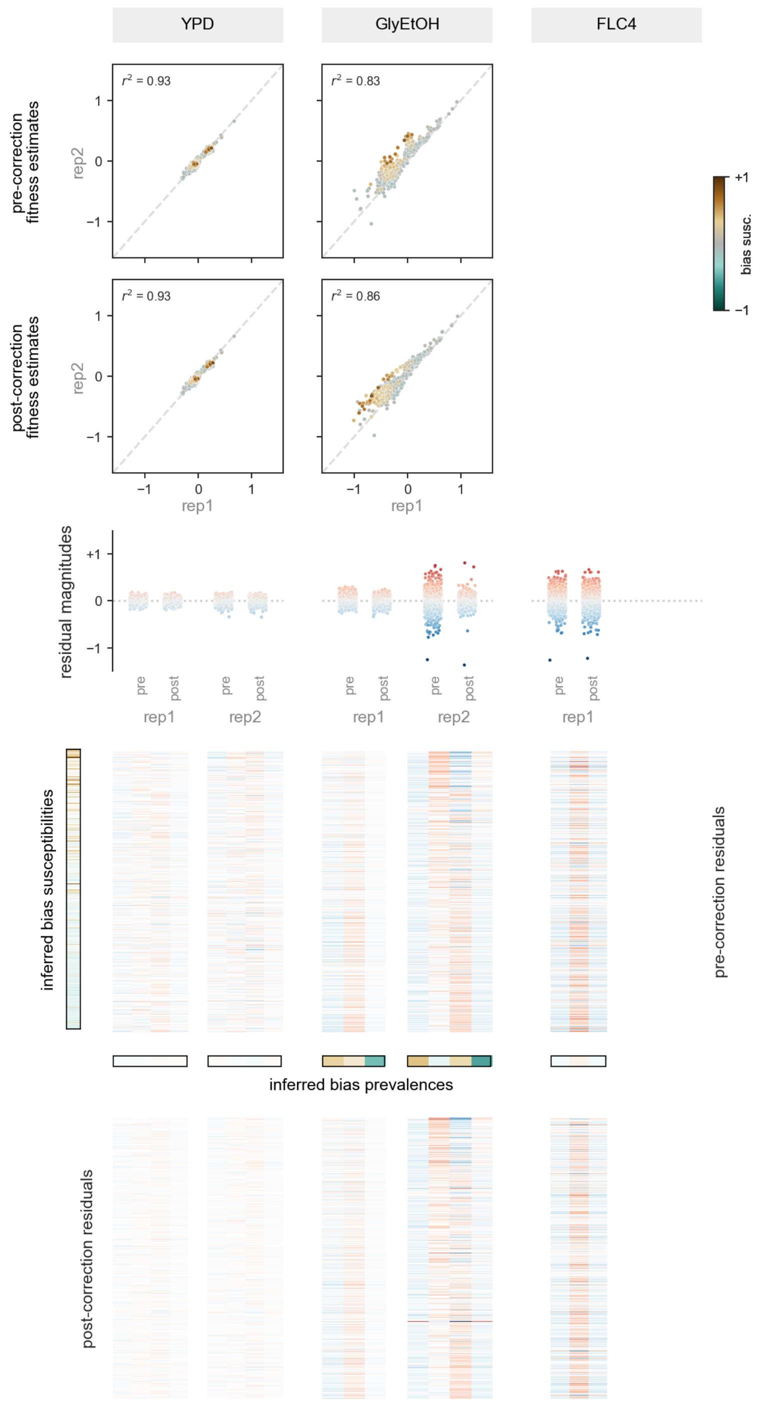

Figure S10: **Results for the Chen et al. (2023) case study (Part 2/2).**
(Scatter plots) Fitness estimates from replicate assays in the same environmental conditions are plotted for all variants (points) before and after bias correction. Variants (points) are colored by the bias susceptibility values inferred by REBAR. **(Jitter plots)** Distributions of residual magnitudes for log-linear fits of variant trajectories are shown for pre-correction (raw) counts as well as post-correction (bias-adjusted) counts (each point represents the residual for one variant-sample; distributions combine data from all replicates). **(Heatmaps)** Red-blue heatmaps show the structure of residuals across all variants (rows, sorted by GC ratio increasing top-to-bottom) and samples (columns) before bias correction (above) and after bias correction (below; residuals color scale as in jitter plots). The vertical brown-teal heatmap to the left shows the inferred bias susceptibility value inferred for each variant, and the brown-teal tiles in between the pre- and post-correction residuals heatmaps depict the inferred bias prevalence for the respective samples (bias color scale in upper scatter plots).

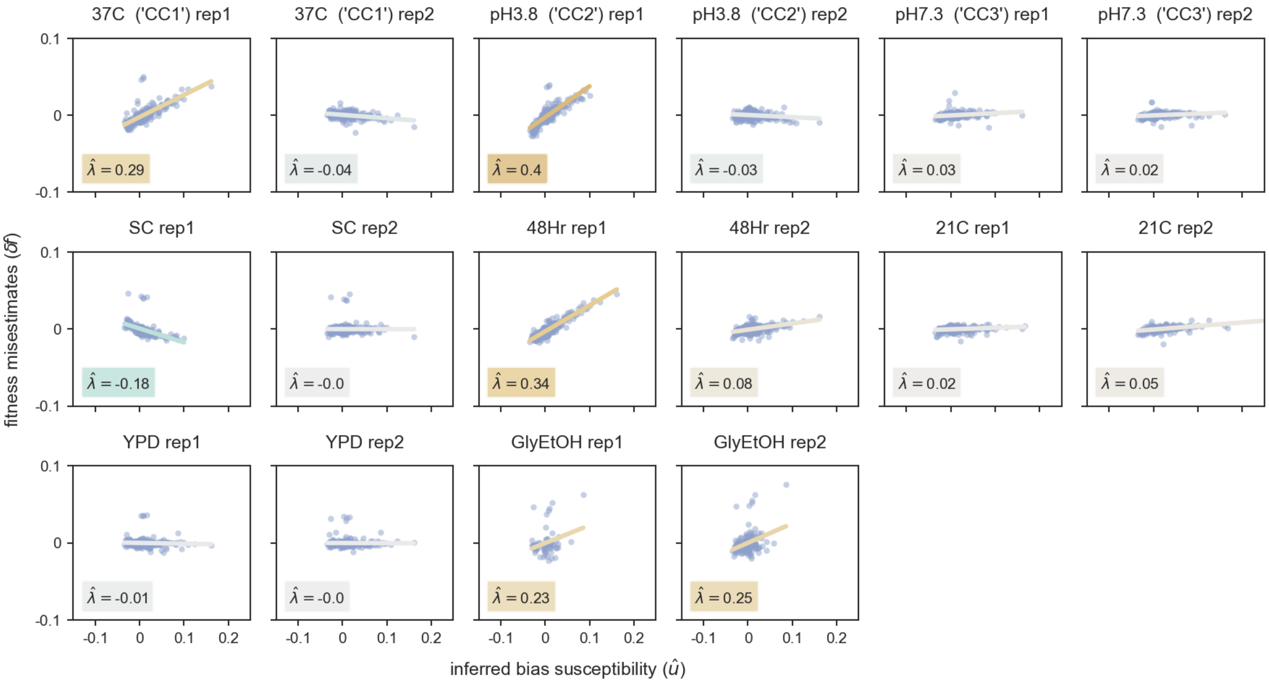

Figure S11: **Inference of bias prevalence trends (Algorithm Stage 2).** The trend in bias prevalence for an assay can be estimated by regressing misestimates of fitness from that assay against bias susceptibilities for a control set of variants for which these values are known (Eq. 3, Supplementary Section S2.2). Each panel presents the results of this regression for one of the 17 assays in the Chen et al. (2023) case study data set. Each point represents a variant in the control set (Supplementary Section S3.2.2). Only control variants that are 'trustworthy' with respect to bias susceptibility inference (Supplementary Section S2.0.1) in a given assay are included in that assay's regression. The best fit line for each assay is shown, the slope of which corresponds to the inferred bias prevalence trend value $\hat{\lambda}^{\alpha}$ for the assay (values listed in the bottom left of each panel). (The lone assay in the FLC4 condition did not have enough trustworthy variants for bias trend inference; therefore its trend value is set to $\lambda^{(\mathrm{FLC4})} = 0$, and it is not shown here).

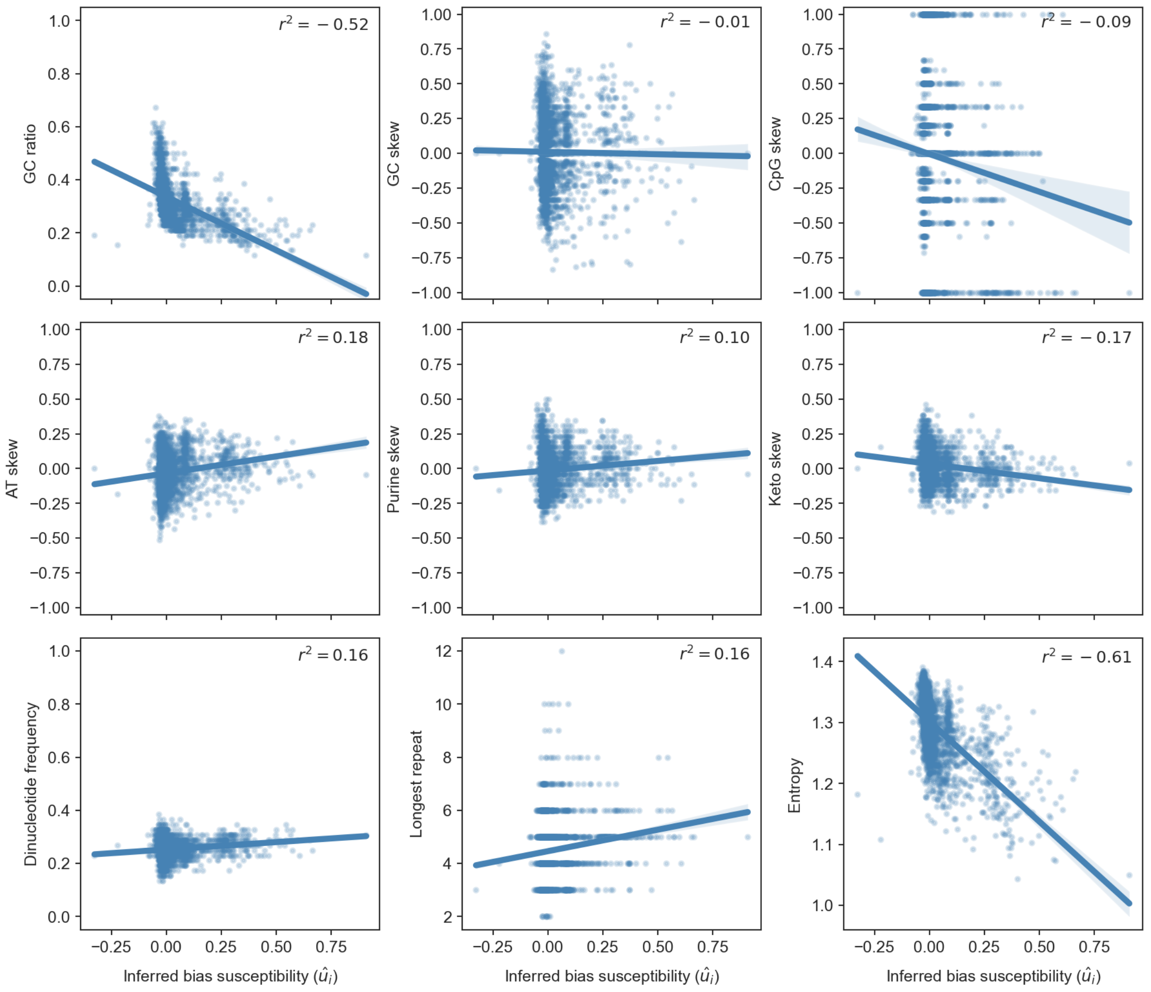

Figure S12: **Correlation of inferred bias susceptibilities with barcode sequence properties.** The bias susceptibility values inferred by REBAR are plotted against various barcode sequence properties for all variants in the Chen et al. (2023) library. The best fit line and coefficient of determination (r^2) are shown for each association. While bias susceptibility appears to be weakly correlated with sequence properties, REBAR can capture and correct its multi-factorial effects.

	Median	Mean	RMS	Std Dev
**High bias conditions (KC1, KC2)**				
Pre-correction fitness estimates ($\bar{f}_i^\alpha$)	0.09	0.12	0.16	0.10
Post-correction fitness estimates ($\tilde{f}_i^\alpha$)	0.06	0.10	0.14	0.10
Fitness corrections ($\Delta f_i^\alpha = \tilde{f}_i^\alpha - \bar{f}_i^\alpha$)	0.02	0.05	0.10	0.09
**All conditions**				
Pre-correction fitness estimates ($\bar{f}_i^\alpha$)	0.07	0.12	0.18	0.13
Post-correction fitness estimates ($\tilde{f}_i^\alpha$)	0.06	0.12	0.18	0.13
Fitness corrections ($\Delta f_i^\alpha = \tilde{f}_i^\alpha - \bar{f}_i^\alpha$)	0.01	0.03	0.06	0.06

Table S3.2.3 Statistics of fitness estimates and fitness corrections for trustworthy variants in the Chen et al. (2023) data set.

S3.3 Sensitivity Analyses

Our REBAR Python package includes functionality for generating synthetic data sets that can be used to test how our method will perform on data sets with properties of interest. Here, we use synthetic data sets to perform sensitivity analyses that evaluate the robustness of REBAR to the quality of the control set and the number of assays in the data set.

S3.3.1 Generation of Synthetic Data

Synthetic data sets with simulated noise and bias effects can be generated using the `generate_synthetic_data()` function of the `rebar.py` module of the REBAR Python package (see `github.com/ryansmcgee/REBAR` for more information about this code and its usage). This function implements simple simulations of bulk fitness assays involving exponential growth, serial transfer, barcode amplification, and sequencing reads for a library of variants.

The user specifies the number of distinct assays to be included in the data set and the time points at which samples will be taken in each assay. Every sample is associated with a ground truth bias prevalence value, which may be specified by the user or set using randomly drawn bias prevalence trend and deviation values (following Eq. S9). The variant library is characterized by a set of ground truth fitness values for each assay as well as a set of ground truth bias susceptibility values (one for each variant). Ground truth bias susceptibility values can be specified explicitly or drawn from a normal distribution with given parameters. In addition, the user designates how many variants are part of the near-equal-fitness control set. These variants are assumed to have normally distributed fitness values with a given mean (0 by default) and standard deviation, which parameterizes the variability of fitness within the control set. The fitnesses of the remaining, non-control variants are specified by the user or drawn from a normal distribution with given parameters.

These ground truth fitness and bias values underlie the generation of synthetic multi-assay barcode read count data using the following procedure:

- For each assay, α:

  1. Compete the library in batch culture with serial transfer, sampling the population for amplification and sequencing at designated time points.

     i. The initial batch culture is Poisson sampled from a large, uniformly distributed library

stock culture to obtain a designated target abundance (or, equivalently, density) per variant (5,000 by default).

ii. The culture abundance of each variant is updated according to exponential growth at its assay-specific rate (i.e., ground truth fitness value) over the interval between time points.

iii. At each time point t, the grown-up culture is Poisson sampled to obtain a sample for barcode amplification and sequencing

iv. The grown-up culture is then Poisson sampled again and 'transferred' such that a new culture is diluted to have the target abundance (i.e., target abundance per variant × number of variants).

v. Return to (ii) and repeat for the designated number of time points.

2. Amplify barcodes for each sample.

i. For each time point t, the number of amplified copies of the barcode associated with variant i, denoted $\mathcal{B}_{i,t}^{\alpha}$, is obtained using

$$\mathcal{B}_{i,t}^{\alpha} = \mathcal{S}_{i,t}^{\alpha} \times 2^p \times e^{u_i v_t^{\alpha}} ,$$

where $\mathcal{S}_{i,t}^{\alpha}$ is the abundance of variant i in the sample from Step 1.iii., 2^p is the assumed amplification from p cycles of PCR (10 cycles by default), and $e^{u_i v_t^{\alpha}}$ is the effect of barcode processing bias on this copy number, which follows from the corresponding ground truth bias susceptibility and bias prevalence values (u_i and v_t^{α}, respectively).

3. Obtain barcode read counts for each sample.

(a) The raw read count of barcode i in sample t is modeled by a Poisson sampling of that barcode's expected number of total reads according to its relative copy frequency:

$$C_{i,t}^{\alpha} = \text{Poisson}\left(R \frac{\mathcal{B}_{i,t}^{\alpha}}{\sum_i \mathcal{B}_{i,t}^{\alpha}} \right) ,$$

where R is the designated total number of reads per sample (1 million by default).

- The collection of raw read counts $\{C_{i,t}^{\alpha}\}$ for all assays, time points, and variants are saved in a data table ready for use by REBAR, as are variant and sample metadata (including ground truth fitness and bias component values for reference).

Note that the raw read count data generated using this procedure incorporate the effects of barcode processing bias consistent with our model (Supplementary Section S1.1), as well as the effects of Poisson noise propagation associated with a plausible sequence of sampling steps.

S3.3.2 Sensitivity to Control Set

In this sensitivity analysis, we vary the size and fitness variance of the control set used by REBAR. We parameterize the control set to have between 0 and 50 variants (making up 0% to 10% of the total 500-variant library) and a fitness variance ranging from 0% (identical variants) to 100% that of the overall library (a fully degenerate 'control set'). For each combination of control set parameters, we run REBAR on 50 different synthetic data sets that were randomly generated using the procedure described in Supplementary Section S3.3.1. Results of this sensitivity analysis are presented in Figure 6. Parameters used for synthetic data generation are listed in Table S3.3.2 below, and parameters for the execution of REBAR are listed in Supplementary Section S3.4.

Parameter	Value
Number of assays	5
Sample time points	$\{0, 1, 2, 3, 4\}$
Total library size	500 variants
Control set size	`VARIED:[0, 10, 20, 30, 40 , 50]`
Ground truth fitnesses, non-control set	Normal($\mu = 0$, $\sigma = 0.3$)
Ground truth fitnesses, control set	Normal($\mu = 0$, $\sigma =$ `VARIED:[0.0, 0.06, 0.12, 0.18, 0.24, 0.30]`)
Ground truth bias susceptibilities (u_i)	Normal($\mu = 0$, $\sigma = 0.1$)
Ground truth bias prevalence trend ($\lambda^{(\alpha)}$)	Normal($\mu = 0$, $\sigma = 0.5$)
Ground truth bias prevalence deviations ($\gamma_t^{(\alpha)}$)	Normal($\mu = 0$, $\sigma = 0.5$)
Target culture abundance per variant	5,000
Number of PCR cycles (p)	10
Total reads per sample (R)	1 million

Table S3.3.2 Parameters used in synthetic data generation for this sensitivity analysis.

S3.3.3 Sensitivity to Number of Assays

In this sensitivity analysis, we vary the number of assays included in the data sets processed by REBAR in order to assess the accuracy of REBAR's fitness estimates as the amount of constraining data decreases. Here we consider synthetic libraries that either a) have a distribution of bias susceptibilities in line with our other case studies, or b) are completely unsusceptible to bias. This allows us to gauge whether or not REBAR overfits noise in the absence of actual bias across data set sizes. For each combination of assay number and bias susceptibility parameters, we run REBAR on 30 different synthetic

data sets that were randomly generated using the procedure described in Supplementary Section S3.3.1. Results of this sensitivity analysis are presented in Figure S13 below. Parameters used for synthetic data generation are listed in Table S3.3.3 below, and parameters for the execution of REBAR are listed in Supplementary Section S3.4.

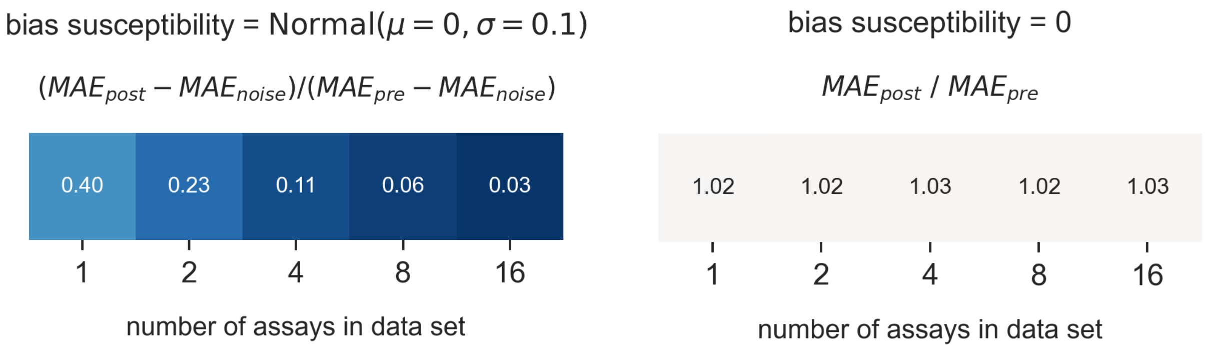

Figure S13: **Sensitivity of fitness error reduction to number of assays in data set.** We tested REBAR on synthetic data sets with simulated noise and bias effects (Supplementary Section S3.3.1). Each synthetic data set includes barcode read counts for a library of 500 variants in a varying number of simulated bulk fitness assays, where ground truth fitnesses, bias susceptibilities, and bias prevalences are assigned randomly. We measure the mean absolute error (MAE) in fitness estimates for each data set before and after correction by REBAR ($\mathrm{MAE}_{\mathrm{pre}}$ and $\mathrm{MAE}_{\mathrm{post}}$, respectively). We also estimate the error in fitness estimates that is expected of Poisson sampling noise alone in the absence of bias for each data set $\mathrm{MAE}_{\mathrm{noise}}$. **(Left)** Here we test REBAR on data sets where variant bias susceptibilities are normally distributed with zero mean and a standard deviation of 0.1, which is consistent with inferred susceptibility values in our other case studies. Each cell in the heatmap reports the median 'relative fitness error after correction' ε for 30 synthetic data sets, where $\varepsilon = (\mathrm{MAE}_{\mathrm{post}} - \mathrm{MAE}_{\mathrm{noise}})/(\mathrm{MAE}_{\mathrm{pre}} - \mathrm{MAE}_{\mathrm{noise}})$. REBAR typically removes greater than 90% of the initial fitness error attributable to bias when used on a data set with 8 or more assays. The amount of error reduction scales approximately linearly with the number of assays, but REBAR can remove 60% or more of bias-induced error when applied to data from just a single assay. **(Right)** Here we test REBAR on data sets where bias susceptibility is 0 for all variants, and thus there are no realized effects from bias. In this setting where fitness errors are only attributable to noise, the 'relative fitness error after correction' metric is not well-behaved, so we instead simply measure the ratio of post-correction error to pre-correction error. We see that This ratio is close to 1 and does not change appreciably as the number of assays in a data set decreases. Therefore, REBAR does not appear to overfit noise in the absence of bias regardless of data set size.

Parameter	Value
Number of assays	`VARIED:[1, 2, 4, 8, 16]`
Sample time points	$\{0, 1, 2, 3, 4\}$
Total library size	500 variants
Control set size	25 variants
Ground truth fitnesses, non-control set	Normal($\mu = 0$, $\sigma = 0.3$)
Ground truth fitness, control set	0
Ground truth bias susceptibilities (u_i)	Normal($\mu = 0$, $\sigma =$ `VARIED:[0.0, 0.1]`)
Ground truth bias prevalence trend ($\lambda^{(\alpha)}$)	Normal($\mu = 0$, $\sigma = 0.5$)
Ground truth bias prevalence deviations ($\gamma_t^{(\alpha)}$)	Normal($\mu = 0$, $\sigma = 0.5$)
Target culture abundance per variant	5,000
Number of PCR cycles (p)	10
Total reads per sample (R)	1 million

Table S3.3.3 Parameters used in synthetic data generation for this sensitivity analysis.

S3.4 REBAR parameterization

In all case studies and sensitivity analyses, we ran REBAR using the following parameters:

Param.	Value	Description	More information
$\theta_{\min}$	300	Minimum mean raw count threshold for variant 'trustworthiness' in a given assay	Supplementary Section S2.0.1
$\theta_{\max}$	∞	Maximum mean raw count threshold for variant 'trustworthiness' in a given assay	Supplementary Section S2.0.1
Φ	0	Minimum number of 'trustworthy' assays per variant for bias susceptibility inference	Supplementary Section S2.0.1
$W_{\max}$	300	Maximum data point weight (for use in weighted regression)	Supplementary Section S2.0.2
μ_u	0	Mean of normal distribution used to draw initial bias susceptibility values	Supplementary Section S2.0.3
σ_u	0.01	Standard deviation of normal distribution used to draw initial bias susceptibility values	Supplementary Section S2.0.3
μ_v	0	Mean of normal distribution used to draw initial bias prevalence values	Supplementary Section S2.0.3
σ_v	0.01	Standard deviation of normal distribution used to draw initial bias prevalence values	Supplementary Section S2.0.3
-	5	Number of Stage 1 inference iterations	Supplementary Section S2.1
η_u	3	Weight of ridge-like penalty term on the magnitude of inferred bias susceptibility values	Supplementary Section S2.1a
η_v	1	Weight of ridge-like penalty term on the magnitude of inferred bias prevalence values	Supplementary Section S2.1b
σ_u^*	0.1	Target standard deviation for inferred bias susceptibility values (used in renormalization)	Supplementary Section S2.2

Table S3.4 Parameterization of our method for the case study analyses.
